# Associations between paracetamol (acetaminophen) intake between 18 and 32 weeks gestation and neurocognitive outcomes in the child: A longitudinal cohort study

**DOI:** 10.1111/ppe.12582

**Published:** 2019-09-15

**Authors:** Jean Golding, Steven Gregory, Rosie Clark, Genette Ellis, Yasmin Iles‐Caven, Kate Northstone

**Affiliations:** ^1^ Centre for Academic Child Health, Population Health Sciences, Bristol Medical School University of Bristol Bristol UK; ^2^ Population Health Sciences, Bristol Medical School University of Bristol Bristol UK

**Keywords:** ALSPAC, child cognitive outcomes, childhood behaviour, exposome, paracetamol, prenatal medication

## Abstract

**Background:**

The majority of epidemiological studies concerning possible adverse effects of paracetamol (acetaminophen) in pregnancy have been focussed on childhood asthma. Initial results of a robust association have been confirmed in several studies. Recently, a few cohort studies have looked at particular neurocognitive outcomes, and several have implicated hyperactivity.

**Objectives:**

In order to confirm these findings, further information and results are required. Here, we assess whether paracetamol intake between 18 and 32 weeks gestation is associated with childhood behavioural and cognitive outcomes using a large population.

**Methods:**

Data collected by the Avon Longitudinal Study of Parents and Children (ALSPAC) at 32 weeks gestation and referring to the period from 18 to 32 weeks, identified 43.9% of women having taken paracetamol. We used an exposome analysis first to determine the background factors associated with pregnant women taking the drug, and then allowed for those factors to assess associations with child outcomes (measured using regression analyses).

**Results:**

We identified 15 variables independently associated with taking paracetamol in this time period, which were used as potential confounders. Of the 135 neurocognitive variables considered, adjusting for the likelihood of false discovery, we identified 56 outcomes for adjusted analyses. Adjustment identified 12 showing independent associations with paracetamol use at *P* < .05, four of which were at *P* < .0001 (all related to child behaviours reported by the mother at 42 and 47 months; eg conduct problems: adjusted mean score + 0.22 (95% confidence interval 0.10, 0.33)). There were few associations with behavioural or neurocognitive outcomes after age 7‐8 years, whether reported by the mother or the teacher.

**Conclusions:**

If paracetamol use in mid‐to‐late pregnancy has an adverse effect on child neurocognitive outcome, it appears to mainly relate to the pre‐school period. It is important that these results be tested using other datasets or methodologies before assuming that they are causal.

**Synopsis**

1 **Study Question**

Does paracetamol consumption at 18‐32 weeks of gestation affect childhood neurocognition or behaviour?

2 **What's already known**

Previous epidemiological studies concerning possible adverse effects of paracetamol (acetaminophen) consumption in pregnancy have suggested associations with cognition, and childhood behaviour, particularly involving hyperactivity and/or attention problems.

3 **What this study adds**

Using a large prospective cohort of 14,062 children, linked to information on child cognitive and behavioural outcomes from 6 months to 17 years, we have shown that paracetamol consumption between 18 and 32 weeks gestation was associated with adverse trends in pre‐school child behaviour, but the associations were no longer present by the end of primary school (age 10‐11 years). Boys appeared to be more susceptible than girls to possible behavioural effects of the drug.

## 1 BACKGROUND

The thalidomide tragedy involved an apparently benign drug being taken for a condition that is common in early pregnancy (nausea and vomiting); its teratogenic effect was discovered because many of the offspring were born with striking and unusual malformations.^1^ Ever since, there has been a concern that relatively common adverse effects would not be easily identified, especially if they were not apparent immediately after the child was born. Few such effects have been discovered, but few drugs taken in pregnancy have been investigated for outcomes that would take many years to be recognised. This is particularly (but not exclusively) true of over‐the‐counter medications, currently the most common of which is paracetamol.

In general, despite evidence that paracetamol crosses the placenta^2^ and is a known endocrine disruptor^3^ and a COX inhibitor,^4^ the medical profession is relatively relaxed about the use of this analgesic in pregnancy; it is usually claimed to be the analgesic of choice. It is true that it has not been implicated in major malformations,^5^ but there has been consistent evidence that women who take paracetamol in pregnancy increase the risk of their child developing asthma.^6^ In addition, there is now increasing evidence that prenatal exposure to the drug increases the risk of childhood hyperactivity and diagnosed ADHD.^7^, ^8^ There is some evidence of an increased risk of autism^9^ but one study found that this was conditional on the autistic child being hyperactive.^10^ In addition, other neurocognitive outcomes have been suggested and these include: various behaviour problems;^11^ poorer attention and executive function;^12^ diagnoses of Oppositional Defiant Disorder (ODD)^13^ and cerebral palsy.^14^ In contrast, there are no reported associations in the offspring of psychotic symptoms^15^ or miscarriage,^16^ and contradictory findings with IQ.^17^, ^18^

Because it is so common, and no links with congenital malformations have been made, paracetamol is considered the analgesic of choice for pregnant women.^19^ It is commonly taken in Europe, with estimates of fetal exposure from over 40% of pregnancies in Spain,^9^ 55% in Denmark^7^ to 65% in France.^20^ When assessing effects of prenatal exposures to the child with a neurocognitive or behavioural problem, maternal recall of events in a pregnancy occurring years earlier, is likely to result in failure to record many common exposures, particularly if she has had more than one pregnancy. Consequently, the most efficient and effective method is to use data collected longitudinally, starting in pregnancy. This we do here by analysing information collected by the Avon Longitudinal Study of Parents and Children (ALSPAC). First, we determined the factors that were independently associated with the intake of paracetamol using an exposome technique,^21^ and then taking these factors into account, we determined independent associations with cognitive and behavioural outcomes of the offspring.

## 2 METHODS

Pregnant women who were resident in Avon, UK with expected dates of delivery between 1st April 1991 and 31st December 1992 were invited to take part in the Avon Longitudinal Study of Parents and Children (ALSPAC).^22^, ^23^, ^24^ The initial number of pregnancies enrolled was 14 541 (for these at least one questionnaire had been returned). Of these initial pregnancies, 14 150 reached 32 weeks (391 having miscarried, been terminated or delivered before this gestation). Questionnaires sent out at this stage included information on paracetamol intake, and were returned by 12 418 (88%) of these women. The study website contains details of all the data that are available through a fully searchable data dictionary and variable search tool: http://www.bristol.ac.uk/alspac/researchers/our-data/. Ethical approval for the study was obtained from the ALSPAC Ethics and Law Committee and the Local Research Ethics Committees (ALEC; IRB00003312) (registered on the Office of Human Research Protections database as U Bristol IRB #1). Informed consent for the use of data collected via questionnaires and clinics was obtained from participants following the recommendations of the ALSPAC Ethics and Law Committee at the time.^25^ Further detail can be found on the study website: http://www.bristol.ac.uk/alspac/researchers/research-ethics/

### 2.1 Exposure

Women in the study were sent a questionnaire entitled ‘Your Pregnancy’ at about 32 weeks gestation. Of the 14 150 women in the study, 12 418 (88%) returned the questionnaire, and 12 025 of these replied to the question on paracetamol use. Data were not available for women who had delivered prior to 32 weeks gestation, and those few who did not enrol in the study until after the baby was born.

The questions asked were: *‘Please indicate how often you have taken the following pills in the last three months’. (a) Aspirin; (b) Paracetamol; (c) Codeine; (d) Mogadon or other sleeping tablets; (e) Valium or other tranquilliser*. Possible responses were as follows: *everyday; most days; sometimes; not at all.* The proportion answering positively to each question (ie *any* use) were as follows: 3.3%; 43.9%; 2.0%; 0.7% and 0.2%, respectively. We only consider the paracetamol responses in this paper. It should be noted that only 0.9% of the study mothers reported taking paracetamol on most days, and only 0.2% every day; the remainder answered ‘sometimes’.

### 2.2 Outcomes

#### 2.2.1 Choice of outcomes

ALSPAC has collected a unique quantity of information on the study offspring, including biological markers, physiological and psychiatric tests as well as details of many neurocognitive measures. In this paper, we have chosen to study two of the domains most mentioned in the literature and summarised in the introduction: measures of cognition and behaviour. We have tested the hypotheses that paracetamol taken in the period between 18 and 32 weeks of pregnancy increases the risk of behaviour problems, particularly hyperactivity and conduct disorder, and we aim to determine the associations with cognition, having no prior hypotheses. We have excluded from consideration at this point associations with educational abilities, speech characteristics, motor development and psychiatric measures of depression or psychosis—they will be considered in subsequent publications.

In order to determine which outcome measures to analyse, we first determined which of 135 outcomes measured on a continuous scale were associated with paracetamol on unadjusted analyses at *P* < 0.0001 (Table 1). To do this, we determined the proportion of each type (ie cognition, temperament and behaviour) and age when the measure was obtained. Firstly, we considered cognitive tests involving IQ and memory. Of 18 measures, 11 (61%) were associated—each involving different measures of IQ. Consideration of the 24 measures of temperament identified associations with 11 (46%) measures, the majority being of the Carey temperament at ages 6 and 24 months.^26^, ^27^ There were 93 measures of behaviour—associations were particularly noted with measures from the Strengths and Difficulties Questionnaire (SDQ)^28^ where 20 of 29 behaviours reported by the mother were associated but only 1 of 12 reported by the teacher; also associated were measures of troublesome behaviour and of activity and attention as reported in the DAWBA set of questions^29^ administered at 7 years. In contrast, the administration of the TEACh tasks of selective attention showed no associations, in contradiction to the findings of the Danish study of a subsample of 5‐year olds.^12^

**Table 1:** Assessment of cognitive and behavioural outcomes considered and the no. (proportion) with unadjusted associations of *P* < .0001 with paracetamol taken between 18 and 32 wk of pregnancy

Measure	Age	No. of tests	No. of tests (%) at *P* < .001
Cognitive tests			
IQ^36^	8 y	6	5 (83)
	15 y	6	6 (100)
Memory^37^	8 y	2	0
	10 y	2	0
	17 y	2	0
Temperament			
Carey temperament^26^, ^27^	6 mo	10	5 (50)
	24 mo	10	5 (50)
EAS temperament^30^	38 mo	4	1 (25)
Behaviour			
Rutter^31^	42 mo	5	4 (80)
SDQ^28^ – mother	47 mo	6	4 (67)
	6 y	6	4 (67)
	9 y	6	4 (67)
	11 y	6	4 (67)
SDQ^28^ – teacher	7‐8 y	6	1 (17)
	10‐11 y	6	0
Feeding difficulties	7 y	1	0
DAWBA^29^ outcomes			
‐ Anxieties	7 y	10	2 (20)
‐ Compulsions	7 y	5	0
‐ Troublesome behaviour	7 y	2	2 (100)
‐ Activity & attention	7 y	9	9 (100)
Selective attention tests^38^	8 y, 11 y	25	0

From these unadjusted analyses, we chose the following domains for detailed analysis: cognition—the IQ test results at ages 8 and 15 (n = 11); temperament—ten Carey and one Emotionality, Activity, and Sociability (EAS)^30^ temperament variables (n = 11); behaviour—the Rutter,^31^ SDQ and DAWBA activity and attention variables (n = 34). Descriptions of the items are in the Supplementary Information.

### 2.3 Statistical analysis

The analyses were undertaken in two strands: (a) a determination of those variables to be included as potential confounders, and (b) an assessment of the relationship between paracetamol and outcome, taking the potential confounders into account; further analyses repeated the analyses for boys and girls separately.

1. The identification of potential confounders used an exposome technique as developed earlier.^21^ This involves: (a) identifying all factors that were associated at *P* < .0001 with paracetamol intake at 32 weeks gestation in unadjusted analyses, (b) putting them into meaningful groups based on physical or psychological attributes, (c) within each group using step‐wise logistic regression to select those variables independently associated with taking the drug, (d) combining the variables identified as independent from each group with those from other groups, then use a step‐wise logistic regression to identify variables to include in a final model. The variables in this model will be those used as potential confounders.
2. Altogether 135 unadjusted relationships between paracetamol intake 18‐32 weeks and the cognitive and behaviour outcomes recorded by ALSPAC were considered. Of these, 41% (n = 56) associated at *P* < .001 were identified for adjusted analysis (Table 1). In instances, where more than one similar outcome were available a decision was made to use the outcome with the highest numbers of children involved.

In general, the outcomes were measured on a continuous scale and thus multivariable linear regression was employed using the raw scores. Since there were no differences between boys and girls in regard to the likelihood that they had been exposed to paracetamol (50.5% and 49.5%, respectively), sex of the child was not used as a confounder. However, in order to determine whether associations differed by sex, stratified analyses were undertaken to determine whether there were apparent interactions—if so, interaction terms would be included in the analyses.

#### 2.3.1 Missing data

We have not modelled using missing data techniques as the data are unlikely to be missing at random. The proportion of missing data varies for each variable, but can be readily ascertained from the ALSPAC study data dictionary (http://www.bristol.ac.uk/alspac/researchers/our-data **);** it varies from 0% for the sex of the child to 62% for the IQ measure at age 15.

## 3 RESULTS

### 3.1 Identification of potential confounders

As noted earlier, 43.9% of the women answering the questionnaire administered at 32 weeks gestation reported taking paracetamol since 18 weeks. In order to identify potential confounders, we considered all features of the mother that were relevant to the period 18‐32 weeks pregnancy or earlier in pregnancy or during the mother's previous life. Of the factors considered, 33 were associated at *P* < .0001 with paracetamol intake, and a further two variables were considered as likely associations even though not associated at this level (mother had rheumatism and social class of partner). The 35 variables were divided into four groups (Table S1):

- background aspects of health (history of the following conditions: hay fever, asthma, indigestion, eczema, back pain, pelvic inflammatory disease, hypertension, migraine, severe depression and rheumatism).
- current health in the period 18‐32 weeks (in poor health, had a cold, had ‘flu, had an infection, had a headache, had backache, high scores on anxiety, malaise and depression scales at 32 weeks). Since there was no obvious category for body mass index (BMI; weight (Kg)/height (m)^2^) pre‐pregnancy, it was included in this group.
- maternal lifestyle (‘healthy’ dietary pattern score and ‘processed’ dietary pattern score,^32^ whether ever smoked regularly, number of cigarettes smoked per day at 32 weeks, any passive smoking, alcohol consumed (none v any), amount of caffeine consumed from beverages).
- social conditions (living in public housing, crowding in home (persons/room), maternal education level, domestic chemical cleaning score,^33^ whether mother worked in pregnancy, age at first pregnancy, parity (no. of previous births) and social class (based on the current occupation of the mother's partner).

Intra‐group analyses resulted in nine variables being eliminated from the following groups: (a) hay fever, eczema and rheumatism; (b) backache and malaise; (c) ever smoked and passive smoking; (d) maternal education and social class. The 15 surviving variables from (a) and (b) were offered together, with the result that pelvic inflammatory disease, hypertension and a history of severe depression were dropped. In parallel, the 11 surviving variables from (c) and (d) were offered together—caffeine consumption, residing in public housing and crowding index were dropped. The 20 surviving variables were then offered together, and five variables were eliminated (anxiety and depression at 32 weeks; no. of cigarettes smoked at 32 weeks; whether the mother worked during pregnancy, and her age at first pregnancy).

The 15 independent predictors of taking paracetamol between 18 and 32 weeks are shown in Table 2. Not unexpectedly, the factor with the largest odds ratio was the report of headache(s) in the 18‐32 week period. This was independent of having a history of migraine. Protective factors were close adherence to eating a ‘healthy’ dietary pattern and of taking no alcohol during this time period. Interestingly, none of the traditional measures of socio‐economic status were retained in the model.

**Table 2:** The potential confounders

Feature	Took drug	Did not take drug	OR (95% CI)	*P*
Medical history				
History of asthma	13.6%	9.9%	1.32 (1.15, 1.52)	1.0 × 10^−4^
History of indigestion	75.7%	66.2%	1.19 (1.08, 1.32)	0.001
History of back pain	52.6%	42.7%	1.11 (1.01, 1.22)	0.028
History of migraine	53.7%	36.5%	1.46 (1.33, 1.60)	5.4 × 10^−16^
Pre‐pregnancy BMI^a^	M 23.3	M 22.6	1.03 (1.02, 1.04)	8.0 × 10^−6^
In period 18‐32 wk				
In poor health^b^	32.8%	18.8%	1.18 (1.12, 1.25)	8.0 × 10^−9^
Had a cold	48.5%	34.3%	1.44 (1.32, 1.59)	7.7 × 10^−15^
Had ‘flu	8.7%	3.8%	1.37 (1.10, 1.70)	0.005
Had other infection	7.1%	3.4%	1.33 (1.19, 1.49)	8.2 × 10^−7^
Had a headache	80.6%	46.4%	3.89 (3.52, 4.29)	5.5 × 10^−160^
Lifestyle				
Healthy diet score^a^	M ‐ 0.11	M + 0.09	0.87 (0.83, 0.92)	2.4 × 10^−8^
Processed diet score^a^	M + 0.07	M ‐ 0.05	1.06 (1.01, 1.11)	0.014
Drank no alcohol	45.0%	53.7%	0.66 (0.60, 0.72)	1.1 × 10^−19^
Social conditions				
Domestic chemical score^a^	M 11.4	M 10.5	1.02 (1.01, 1.03)	0.002
Parity 1+	62.6%	49.4%	1.18 (1.12, 1.24)	6.2 × 10^−11^

Features of the mother that independently predicted whether she had taken paracetamol in the period 18‐32 weeks. Final model – N = 9522; pseudo‐*R* ^2^ = 14.35%. Details of variables are given in the Supplementary Material.

BMI, body mass index; CI, confidence interval; M, mean; OR, odds ratio or mean difference.

a Odds ratio for a continuous scale.

b Subjective measure reported by the mother at 32 wk.

The 15 variables were treated as possible confounders, since we did not consider it warranted to carry out similar modelling procedures for each of the 56 outcomes and thence identify factors predictive of both paracetamol intake and each outcome. The confounders identified in this way would necessarily include some of the factors predictive of paracetamol intake.

### 3.2 Availability of potential confounders

The 15 variables comprising the final potential confounder model were obtained from four different questionnaires administered during pregnancy. Not every woman completed all questionnaires or questions, consequently data concerning all 15 factors were available for only 9522 (79%) of the 12 025 mothers who completed the paracetamol question in the 32‐week questionnaire. The differences concerning those who were perforce excluded from later analyses are shown by outcome measure in Table S2. In general, confining the analyses by paracetamol to the cases where all potential confounder information is available results in the loss of 15%‐17% of children concerning the scales answered by the parent or resulting from testing in the ALSPAC clinic (eg IQ at 8) and 20% of the teacher reports.

### 3.3 Adjusted analyses for cognition variables

Of the 18 measures of cognitive function considered, the eleven relating to IQ were considered further; almost all subtests and overall measures showed strong inverse unadjusted associations (Table S3). However, on allowing for the 15 potential confounders, although all measures continued to show inverse associations, the effects were largely attenuated: only one showed a significant trend—the ‘Freedom from distractibility’ subset of IQ measured at age 8 (mean difference (MD) −0.35 (95% CI −0.70, −0.00)) (Table 3). Stratification by sex of the child showed no differences of note (Tables 4, 5 and S3a,b).

**Table 3:** Mean differences in outcome measures associated (*P* < .05) with paracetamol exposure at 18‐32 wk gestation

Measure	N^a^	AMD (95% CI)	*P*_trend_
Cognition			
IQ: Freedom from distractibility at 8 y	5448	−0.35 (−0.69, −0.00)	0.048
Temperament			
M.Adaptability at 6 mo	8166	+0.33 (+0.05, +0.60)	0.019
M.Persistence at 24 mo	8074	+0.36 (+0.12, +0.60)	0.003
Behaviour			
M.SDQ Hyperactivity at 42 mo	7849	+0.16 (+0.07, +0.25)	3.3 × 10^−4^
M.SDQ Hyperactivity at 47 mo	7508	+0.22 (+0.10, +0.33)	1.8 × 10^−4^
T.DAWBA Attention at 7‐8 y	4378	+0.45 (+0.11, +0.79)	0.009
T.DAWBA Attention/Activity at 7‐8 y	4376	+0.53 (+0.02, +1.04)	0.042
M.SDQ Conduct problems at 42 mo	7849	+0.22 (+0.10, +0.33)	1.9 × 10^−4^
M.SDQ Conduct problems at 47 mo	7508	+0.08 (+0.01, +0.15)	0.023
M.SDQ Conduct problems at 81 mo	6742	+0.10 (+0.02, +0.18)	0.010
M.SDQ Total behavioural difficulties^b^ at 42 mo	7849	+0.54 (+0.26, +0.82)	1.3 × 10^−4^
M.SDQ Total behavioural difficulties^b^ at 47 mo	7508	+0.31 (+0.08, +0.53)	0.007

For definitions of DAWBA and SDQ see Supplementary Material; the IQ tests were performed by ALSPAC psychologists; the behaviour measures were reported by mothers (labelled ‘M’) within self‐completion questionnaires, with the exception of those labelled ‘T’ which were completed by teachers. Details of outcome measures are given in the Supplementary Material.

AMD, adjusted mean difference of score.

a Adjusting for all factors in Table 2.

b Combination of hyperactivity, conduct, emotional and peer problems.

**Table 4:** Among daughters, mean differences in outcome measures associated with paracetamol exposure at 18‐32 wk gestation

Measure	N^a^	AMD (95% CI)	*P*_trend_
Cognition			
IQ: Freedom from distractibility at 8 y	2726	−0.41 (−0.88, +0.06)	0.084
Temperament			
M.Adaptability at 6 mo	3933	+0.215 (−0.19,+0.62)	0.293
M.Persistence at 24 mo	3883	+0.36 (+0.02, +0.70)	0.039
Behaviour			
M.SDQ Hyperactivity at 42 mo	3781	+0.17 (+0.05, +0.30)	0.006
M.SDQ Hyperactivity at 47 mo	3613	+0.25 (+0.09, +0.41)	0.002
T.SDQ Hyperactivity at 7‐8 y	2177	+0.21 (+0.02, +0.40)	0.033
T.DAWBA Attention at 7‐8 y	2181	+0.66 (+0.27, +1.04)	0.001
T.DAWBA Attention/Activity at 7‐8 y	2484	−0.11 (−0.41, +0.19)	0.478
M.SDQ Conduct problems at 42 mo	3781	+0.16 (−0.00, +0.32)	0.050
M.SDQ Conduct problems at 47 mo	3613	+0.06 (−0.04, +0.16)	0.273
M.SDQ Conduct problems at 81 mo	3266	+0.08 (−0.03, +0.19)	0.169
M.SDQ Total behavioural difficulties^b^ at 42 mo	3781	+0.49 (+0.11, +0.88)	0.012
M.SDQ Total behavioural difficulties^b^ at 47 mo	3613	+0.26 (−0.05, +0.57)	0.100

Outcome measures were either included in Table 3, or have shown effects at *P* < .05. Details of outcome measures are given in the Supplementary Material.

For definitions of DAWBA and SDQ see Supplementary Material; the IQ tests were performed by ALSPAC psychologists; the behaviour measures were reported by mothers (labelled ‘M’) within self‐completion questionnaires, with the exception of those labelled ‘T’ which were completed by teachers.

a Adjusting for all factors in Table 2.

b Combination of hyperactivity, conduct, emotional and peer problems.

**Table 5:** Among sons, mean differences in outcome measures associated with paracetamol exposure at 18‐32 wk gestation

Measure	N^a^	AMD (95% CI)	P_trend_
Cognition			
IQ: Freedom from distractibility at 8y	2722	−0.27 (−0.79, +0.24)	0.249
Temperament			
M.Adaptability at 6m	4233	+0.44 (+0.06, +0.82)	0.037
M.Approach at 6m	4231	+0.44 (+0.03, +0.86)	0.037
M.Distractibility at 6m	4234	+0.40 (+0.02, +0.78)	0.040
M.Difficult baby at 6m	4537	+1.35 (+0.15, +2.54)	0.027
M.Persistence at 24 m	4191	+0.36 (+0.03, +0.69)	0.032
Behaviour			
M.SDQ Hyperactivity at 42m	4068	+0.17 (+0.04, +0.29)	0.010
M.SDQ Hyperactivity at 47m	3895	+0.19 (+0.03, +0.36)	0.019
T.DAWBA Attention at 7‐8 y	2197	+0.29 (−0.24, +0.81)	0.286
T.DAWBA Attention/Activity at 7‐8 y	2195	+0.34 (−0.49, +1.16)	0.427
M.SDQ Conduct problems at 42m	4068	+0.29 (+0.12, +0.45)	0.001
M.SDQ Conduct problems at 47m	3895	+0.10 (+0.00, +0.20)	0.047
M.SDQ Conduct problems at 81m	3476	+0.12 (+0.01, +0.23)	0.027
M.SDQ Conduct problems at 115m	3142	+0.12 (+0.01, +0.24)	0.039
M.SDQ Total behavioural difficulties^b^ at 42m	4068	+0.61 (+0.21, +1.00)	0.003
M.SDQ Total behavioural difficulties^b^ at 47m	3895	+0.35 (+0.03, +0.67)	0.031
M.SDQ Total behavioural difficulties^b^ at 81m	3470	+0.37 (+0.01, +0.73)	0.044

Outcome measures which either were included in Table 3, or which have shown adjusted effects at *P* < .05. Details of outcome measures are given in the Supplementary Material.

For definitions of DAWBA and SDQ see Supplementary Material; the IQ tests were performed by ALSPAC psychologists; the behaviour measures were reported by mothers (labelled ‘M’) within self‐completion questionnaires, with the exception of those labelled ‘T’ which were completed by teachers.

a Adjusting for all factors in Table 2.

b Combination of hyperactivity, conduct, emotional and peer problems.

### 3.4 Adjusted analyses for temperament variables

There were 11 measures of the child's temperament showing a significant unadjusted association with maternal paracetamol intake. On adjustment, two trends retained significance: adaptability (MD 0.33 (95% CI 0.05, 0.60)) at 6 months and persistence (MD 0.36 (95% CI 0.12, 0.60)) at 24 months (Tables 3 and S4). Stratification by sex showed that for boys, there were five temperaments that were not attenuated: approach (MD 0.44 (95% CI 0.03, 0.86)), adaptability (MD 0.44 (95% CI 0.06, 0.82), distractibility (MD 0.40 (95% CI 0.02, 0.78)) and measure of a difficult baby (MD 1.35 (95% CI 0.15, 2.54)) all at 6 months, and persistence at 24 months (MD 0.36 (95% CI 0.03, 0.69)); for girls only persistence at 24 months was retained (MD 0.36 (95% CI 0.02, 0.70)) (Tables 4, 5 and S4a,b). None of the interactions between the sexes were statistically significant.

### 3.5 Adjusted analyses for hyperactive behaviour

Measures of attention and excessive activity used the Strengths and Difficulties Questionnaire (SDQ) measures^28^ at various ages from 3 to 11 years including both maternal and teacher reports, as well as from the adapted Development and Well‐being Assessment (DAWBA) series of questions answered by mother and teacher at age 7‐8.^29^ Of the seven measures of hyperactivity using the SDQ type questions, only two survived adjustment—hyperactivity identified in her child by the mother at ages 42 and 47 months (Table 3). There were no teacher associations, although the measure in school year 3 was of borderline‐adjusted association (MD 0.16 (95% CI −0.01, +0.33)). When the boys and girls were considered separately (Table S5a,b), there were similar significant adjusted associations for hyperactivity at ages 42 and 47 months, and for girls, the teacher report at seven was also significant (Tables 4 and 5).

The DAWBA scales separated hyperactive and attentive behaviour. Of the 13 measures investigated for all children, all of the measures of activity were attenuated, but the attention measures were more likely to have survived adjustment, at least at the <0.10 level. The most relevant adjusted score was for attention as assessed by the teacher in school year 3 (MD 0.45 (95% CI 0.11, 0.79)). There were no such associations for the teachers' report for pupils in school year 6 (ages 10‐11) (Table S6). When boys were considered separately, all the associations were attenuated, but among the girls, there were adjusted associations with both the attention score (MD 0.66 (95% CI 0.27, 1.04)) and a combination of the attention and activity scales (MD 0.79 (95% CI 0.27, 1.32)).

### 3.6 Conduct/troublesome behaviour

A scale for identification of conduct problems was included in the SDQ and in two scales included in the DAWBA as asked of the mother and the teacher. There were adjusted associations evident for the children aged 42, 47 and 81 months (Table 3). When the two sexes were analysed separately, it can be seen that for girls all the associations were attenuated (Table 4) but, for boys, associations were apparent with the SDQ scale at ages 42, 47, 81 and 115 months (Table 5). There were no significant differences between the associations for the two sexes (Tables 4, 5 and S7a,b).

### 3.7 Emotional behaviour

As with the other scales based on the SDQ or Rutter, seven ages were assessed—five by mothers and two by teachers. All were attenuated on adjustment (Tables S8a,b).

### 3.8 Total behavioural difficulties

Summation of the hyperactivity, conduct, peer and emotional difficulty scales of the SDQ was used to create a score of total behavioural difficulties. Although all seven measures were associated with maternal paracetamol intake in pregnancy, the associations were attenuated for all except the two early measures (Table 3). Stratification by sex showed associations on adjustment with the three earliest measures for boys, and the 42‐month measure for girls (Tables 4 and 5 and S9a,b). In all these instances, the child whose mother had taken paracetamol had more behavioural problems.

## 4 COMMENT

### 4.1 Principal findings

In this paper, we have investigated 135 continuous outcomes related to cognition, temperament and behaviour in children born to women who had taken paracetamol at some stage between 18 and 32 weeks of pregnancy compared with those who had not. In all, 56 (41%) were associated at the 0.1% level before adjustment. In order to determine appropriate confounders, we used a hypothesis‐free exposome technique to identify 15 features of the mothers that independently predicted whether she had taken paracetamol. We then used multiple regression to assess the level of association between paracetamol intake and each of the 56 outcomes after adjustment for the 15 potential confounders; after adjustment 12 of the 56 remained significantly associated. The 12 variables were almost entirely features of hyperactive or attentive behaviour—being less adaptable at 6 months, having poorer persistence at 24 months, having elevated scores on the hyperactive behaviour scales at 42 and 47 months (maternal reports) and teacher reports of poor attention in school year 3 (age 7‐8 years). Hands‐on testing to determine IQ at age 8 identified a subcategory ‘freedom from distractibility’ that has an association with attention.^34^ The other factors associating with paracetamol were conduct problems at ages 42 and 47 months, and total behaviour difficulties (which include hyperactivity) at these ages.

This is not the first time that fetal paracetamol exposure has been linked to hyperactivity, but it is probably the first time that the potential confounders have been identified using an exposome technique; this technique was hypothesis‐free, and thus likely to identify some factors that had not previously been considered to be confounders. One other study has used the ALSPAC data to investigate associations between maternal paracetamol intake in pregnancy (both pre‐18 weeks and between 18 and 32 weeks) in regard to hyperactivity and other behaviour measures (using the SDQ) at 81 months. They showed significant associations, particularly with taking the drug in the period 18‐32 weeks and children with the worst levels of hyperactivity, conduct disorder, emotionality and total behaviour difficulties. Their strategy included taking a number of confounders into account, but also assessing whether paternal consumption of paracetamol, or intake by the mother 5 years later showed similar associations.^11^ Our analysis also using these outcomes at 81 months showed that there was an association with conduct disorder, none with emotional symptoms and associations of borderline significance only with hyperactivity and total behavioural difficulties after allowing for the 15 potential confounders (Tables 3 and S4‐S9). These differences could have been because of the different choices of factors adjusted for, or a genuine difference between the dichotomy of identifying the worst 10% on the different scales.

Results from other longitudinal studies concerning neurocognitive outcomes have been reviewed by Bauer and colleagues in 2018.^35^ They identified nine studies from five cohorts, the Norwegian MoBA and the Danish DNBC being the largest and most informative. There were no two studies that measured the exposure, the confounders or the outcomes in the same way. Nevertheless, the conclusion of the review was that ‘associations were strongest for hyperactivity and attention‐related outcomes’. This supports our findings.

In order to determine the possible associations of an environmental exposure with an outcome X, a major task is to ensure that the analyses have appropriately adjusted for factors that are associated with the exposure. Here, we have used an exposome technique to model all available ALSPAC data concerning the study mother prior to 32 weeks gestation to identify the environmental features that need to be taken into account when analysing consequences of the mother's consumption of paracetamol between weeks 18 and 32 of gestation.

The following steps were undertaken: (a) unadjusted assessment of all environmental features that concerned the woman prior to and during pregnancy up until the date on which paracetamol was collected at 32 weeks; (b) selection from (a) of 33 associations with *P* < .0001, and added inclusion of two variables thought to be important—rheumatism and social class; (c) Division of these factors into logical groups; (d) step‐wise logistic regression within each group; (e) combination of the results from the groups to create a final model containing 15 variables with a pseudo‐*R* ^2^ of 14.35%. Interestingly, the modelling exercise (which was carried out using a hypothesis‐free approach) rejected many of the features that were used as confounders by other studies such as use of other medications, a history of smoking, maternal age, socio‐economic status, maternal education level and psychiatric illness.

We repeated the analyses separately for the two sexes and found that the associations with hyperactive and conduct behaviour were more likely to be found among the boys where there were 14 significant adjusted associations (Table 5), in contrast with the girls with only six significant adjusted associations (Table 4). The patterns of association are somewhat different: for boys (but not girls) there is a predominance of difficult temperaments at 6 months, and of associations with conduct disorder from 42 months to 9 years, and of total behavioural difficulties from 42 to 81 months. In contrast, the girls are more likely than the boys to be implicated with attention/hyperactive behaviour especially at older ages (7‐8 years) when reported by the teacher.

It is noteworthy that the literature review of Bauer and colleagues^35^ did not find results of neurocognitive associations with prenatal paracetamol intake beyond the age of 7. In the present study, we have shown that the strongest relationships with child behaviour were identified at 42 and 47 months, and that the SDQ associations diminished as the children became older—this being especially true of the boys. We showed that, although others have shown an association with IQ in the DNBC, this was at age 5. We found only a marginal association with one subgroup at age 8, and none at age 15. This raises a possibility that if paracetamol does have an adverse effect on child temperament and behaviour, this may be confined to the pre‐puberty ages.

### 4.2 Strengths of the study

A strength of this study lies in the fact that it concerns a geographically defined population of pregnant women, not selected in any way other than by the place of residence (Avon) and the expected date of delivery (April 1991 to December 1992). Nevertheless, although about 80% of the eligible population took part, those who did not enrol were more likely to be young, to smoke and be from the more deprived social groups. As we have shown, however, these factors were not linked to the likelihood of the woman taking paracetamol in the period 18‐32 weeks gestation, and consequently may not be responsible for bias.

Another advantage lies in the fact that the study collected information from a variety of sources including hands‐on assessments (eg IQ at 8; the TEACh measures of attention), structured questionnaires completed by the mother (temperament and behaviour), and the teacher (behaviour). Thus, if there were particular biases with the source of data this should be revealed; in fact, most of the positive findings were from maternal report, and the independent hands‐on test results were not related to maternal paracetamol intake. This may, however, be a function of the cognitive test rather than revealing a defect in the design.

### 4.3 Limitations of the data

There are a number of limitations. (a) Although we took time to investigate possible confounders using an exposome technique, the results are necessarily confined to the information collected. There may be many other features that should have been adjusted for (residual confounding), and which may change the conclusions of the paper. (b) Relevant to this is the fact that we used a very statistically stringent approach to identifying the confounders from the data collected by ALSPAC—relaxing the *P* values used for selection may have resulted in a wider list of confounders. (c) It is important to emphasise that we examined paracetamol between 18 and 32 weeks only (but not in early or late pregnancy). (d) We captured some information about frequency of paracetamol use but the numbers of frequent users were too small for further analyses. (e) Importantly, our analysis concentrated on using the continuous data collected on the outcomes considered; this may result in the failure to recognise strong effects in the tail(s) of the distribution.

### 4.4 Interpretation

Unlike most of the other studies (see review by Bauer et al^35^), we have used the continuous trait scales rather than a dichotomy identifying pathology. This gives the study more statistical power but, on the other hand, may hide important associations.

### 4.5 Conclusions

We have shown that paracetamol taken in the period 18‐32 weeks is associated with aspects of child attention and hyperactivity until 7 years of age, but there is little sign of adverse associations at later ages, with the exception of their sons who are more likely to demonstrate conduct problems up until 9 years of age. Given the increase in these behaviours it will be important to assess whether they are accompanied by difficulties in scholastic achievements, or whether any adverse effects survive puberty.

## CONFLICT OF INTEREST

The authors confirm they have no conflicts of interest.
