# Supplementary material for: Associations between paracetamol (acetaminophen) intake between 18 and 32 weeks gestation and neurocognitive outcomes in the child: A longitudinal cohort study
Source: Paediatr Perinat Epidemiol. 2019 Sep 15;34(3):257–66. doi: 10.1111/ppe.12582 (PMC7217049; doi:10.1111/ppe.12582)
Supplement: Supplementary file 1 [file PPE-34-257-s001.docx]

**Supplementary information**

*Cognition*

IQ at age 8

IQ was measured at age 8 using the UK version of the WISC^1^ using the 10 sub-tests: five performance (picture completion, coding, picture arrangement, block design and object assembly) and five verbal (information, similarities, arithmetic, vocabulary and comprehension). A short form of the measure was employed where alternate items (always starting with item number 1 in the standard form) were used for all sub-tests, with the exception of the coding sub-test which was administered in its full form.

In addition to the final IQ scores (i.e. full, performance and verbal), which are the standard measures derived from an IQ test, we used two of the factor-based index scores: (a) Verbal Comprehension Index, calculated using the Information, Similarities, Vocabulary and Comprehension sub-tests; and (b) Perceptual Organisation Index, using the Picture Completion, Picture Arrangement, Block Design and Object Assembly sub-tests. The Freedom from Distractibility Index is the sum of scores on the Arithmetic and Digit Span subtests. Although it has some association with other measures of attention, the associations are said to be weak.^2^

IQ at age 15

(Please note that the description below is largely copied from McCrimmon and Smith^2^)

The Wechsler Abbreviated Scale of Intelligence, Second Edition (WASI-II^4^) updated abbreviated measure of cognitive intelligence designed for individuals 6 to 90 years of age. It was developed to quickly and accurately estimate cognitive intelligence when administration of a full battery is not feasible or necessary.

Although the full WASI-II consists of four sub-tests, selected as those with the highest factor loadings on *g*, or general intelligence. Specifically, Vocabulary and Similarities form the Verbal Comprehension Index (VCI) while Block Design and Matrix Reasoning form the Perceptual Reasoning Index (PRI), which replace the Verbal Intelligence Quotient (VIQ) and Performance Intelligence Quotient (PIQ) from the original WASI, respectively. In addition, all four sub-tests are combined to form the Full-Scale IQ-4 sub-tests (FSIQ-4) while Vocabulary and Matrix Reasoning are combined to form the Full-Scale IQ-2 Sub-tests (FSIQ-2). Because of time and cost restraints just two sub-tests were administered in ALSPAC: the vocabulary and matrix reasoning subtests, which combined form the Full-Scale IQ-2.

*The Vocabulary Sub-test* measures an examinee’s word knowledge, verbal concept formation, fund of knowledge, crystallized intelligence, and degree of language development. It consists of 31 items, including three initial picture items. Examinees are required to verbally define and/or describe a word or concept that is orally presented to them by the examiner (participants are also provided a Stimulus Book which presents each word in a written format). All participants begin with item four, and reverse back to the pictured items if necessary. Scoring for each item is done on a zero-, one-, or two-point basis according to the general scoring principles outlined in the manual. In addition, the manual outlines specific zero or one point responses to be queried by the examiner to garner additional detail or clarity. The sub-test is discontinued after three consecutive failures.

*Matrix Reasoning* measures fluid and visual intelligence, spatial ability, and perceptual organization. It consists of 30 visually-depicted incomplete matrices presented in the Stimulus Book. Examinees are required to view each incomplete matrix and choose one item from a selection of five options at the bottom of each page that correctly completes the matrix. Each correct item receives one point. The sub-test is discontinued after three consecutive failures.

The WASI-II has several features that make it a measure of choice for the brief evaluation of intelligence. It has a strong historical background in the Wechsler tradition and is suitable for clinical and research applications. It is normed on a large and representative sample and is appropriate for a broad age range. It has both four and two subtest options, providing examiners with the option when selecting a brief measure of intelligence. The ceiling and floor of each subtest has been extended, allowing for enhanced evaluation of intelligence at the upper and lower ends. The administration instructions and discontinue and reversal rules have been simplified and reduced. It is strongly linked to both the WISC-IV and WAIS-IV, allowing for substitution of sub-test scores with those more comprehensive measures. Moreover, it has been linked to measures of achievement, allowing for comparisons between measures.

*Carey Temperament Scales*

There are two scales, known as the Carey Temperament scales, one asked in the questionnaire sent to the mother at 6 months and the other at 2 years. Although the questions are slightly different, there are algorithms to calculate nine different scales at each age.

When the infant was 6 months old an adaptation of the revised infant temperament questionnaire (RITQ)^5^ was used. RITQ is a valid and reliable measure of temperament.^5^ The adapted RITQ used in ALSPAC comprised 88 of the 95 original questions in the RITQ. Omitted from the original version of 99 questions were those questions which on piloting showed a non-response from at least 10% of the population. In general, these depended on the presence of certain characteristics. For example, a question on the baby's reactions when having an infection was irrelevant to those mothers whose baby had never had an infection. The internal consistency of the 88-item RITQ ranged from 0.40 to 0.73 for sub-scales, and 0.79 for the composite score. This is consistent with the internal consistencies of the original questions included in the RITQ.^6^ A detailed study has compared the norms reported by Carey and McDevitt,^6^ which used only a small number of infants in the USA, with those of the ALSPAC study with its considerably larger numbers.^8^ ALSPAC derived a prorated score for each 6-month temperament corrected for age at completion of the questionnaire and gestation at delivery, as the raw scores were found to be highly dependent on these variables.

In the **6-month** questionnaire the rubric prior to the first question was worded as follows: ‘*These questions are about how your baby behaves. Although some of them seem similar to one another, please answer them all. How often has the baby's recent behaviour been like the following descriptions?’* The options given for each question were: ‘*almost never; rarely; usually does not; usually does; often; almost always’.* The 88 questions were included in the 10 different temperament scores as devised by Carey. These are as follows:

Activity: This was derived from 12 questions, such as: *He moves much and for several minutes or more when playing by himself (kicking, waving arms and bouncing).* High scores indicate most active.

Rhythmicity: was derived from 10 questions including: *He wants an extra feed at a different time each day (over one hour difference).* High scores indicate less rhythmic (regular).

Approach: This is derived from 10 questions such as: *His first reaction to any new procedure (first haircut, new medicine, etc) is objection.* High scores indicate most difficult behaviour.

Adaptability: derived from 10 questions such as: *He requires introduction of a new food on three or more occasions before he will accept (swallow) it.* High scores imply less adaptable.

Intensity was derived from 10 other questions. Examples are: *He reacts strongly to strangers: laughing or crying;* and *He displays much feeling (vigorous laughing or crying) during nappy change or dressing.* High scores indicate greater intensity of responses.

Mood score was calculated from nine questions such as *He cries when left to play alone.* The higher the score the worse the mood.

Persistent temperament was calculated from seven questions, for example *He plays with a toy for less than a minute and then looks for another toy or activity.* The score increased for poorer persistence.

Distractibility: this score was derived from 10 questions such as - *He continues to reject disliked food or medicine in spite of your efforts to distract with games or tricks.* Highest scores indicate easily distracted.

Threshold: the score was calculated from 10 questions such as - *He reacts even to a gentle touch (is startled, wriggles, laughs, cries).* The higher the score the more reactive he was (i.e. the lower the threshold).

**At 24 months,** 89 items were adapted from the Carey Toddler Temperament Scale.^8^ The same nine sub-scales were derived from these scales.

The rubric prior to the first question was worded as follows: ‘*The next questions are important. They will tell us about his personality. Some questions look similar but are not. Please tick the box which describes how often your son behaves like the description’.* The options given for each question were: ‘*almost never; rarely; sometimes; often; almost always’.*

Activity: This was derived from nine questions, such as: *He moves much and for several minutes or more when playing by himself (kicking, waving arms and bouncing).* High scores indicate most active.

Rhythmicity: was derived from 11 questions including: *He wants an extra feed at a different time each day (over one hour difference).* High scores indicate less rhythmic (regular).

Approach: This is derived from 11 questions such as: *His first reaction to any new procedure (first haircut, new medicine, etc) is objection.* High scores indicate most difficult behaviour.

Adaptability: derived from seven questions such as: *He requires introduction of a new food on 3 or more occasions before he will accept (swallow) it.* High scores imply less adaptable.

Intensity was derived from nine other questions. Examples are: *He reacts strongly to strangers: laughing or crying;* and *He displays much feeling (vigorous laughing or crying) during* nappy change or dressing. High scores indicate greater intensity of responses.

Mood score was calculated from 12 questions such as *He cries when left to play alone.* The higher the score the worse the mood.

Persistent temperament was calculated from 10 questions, for example *He plays with a toy for less than a minute and then looks for another toy or activity.* The score increased for poorer persistence.

Distractibility: this score was derived from 10 questions such as - *He continues to reject disliked food or medicine in spite of your efforts to distract with games or tricks.* Increasing scores indicate that the child is distracted easily.

Threshold: the score was calculated from 8 questions such as - *He reacts even to a gentle touch (is startled, wriggles, laughs, cries).* The higher the score the more reactive he was.

From these scores a Difficult child score was derived using the scales as identified by Fullard et al,^8^ comprising high activity, low approach, low adaptability, low persistence, high intensity and negative mood.

***The EAS Temperament scale***

Mothers completed the Emotionality Activity Sociability Temperament Survey for Children^9^ at 38 months. Twenty statements assessing four scales (five items each) were each rated on a 5‐point Likert‐type scale (1 = child's behaviours are *not at all like* this; 5 = child's behaviours are *exactly like* this). The four scales corresponded to traits described by Buss and Plomin^9^ as Activity (preferred level of activity, e.g., ‘is very energetic’), Emotionality (tendency to show distress, e.g., ‘often fusses and cries’), Shyness (tendency to be inhibited with unfamiliar people, e.g., ‘takes a long time to warm up to strangers’), and Sociability (tendency to prefer the company of others, e.g., ‘likes to be with people’). Items were summed (after reverse coding for items worded in reversed direction) with higher scores indicating greater appraised levels of the trait being measured. The EAS has good psychometric properties of reliability and validity and has been translated into a variety of languages (see Bornstein et al^10^ for references). Across three data collection waves, Bornstein et al^10^ reported internal consistency (α) estimates in the current sample ranged from .77 to .78 on the Activity scale, all were .84 on the Emotionality scale, ranged from .78 to .83 on the Shyness scale, and ranged from .61 to .63 on the Sociability scale.

***Behaviour***

*The Revised Rutter Scale*

The Revised Rutter Parent Scale for Preschool Children is an extension of the Rutter

behaviour scale.^11^ It comprised 29 items and was completed by the chief carer at 42 months of age. The scale measures four different domains: hyperactivity (four items), emotional difficulties (six items), conduct difficulties (eight items) and pro-social behaviours (11 items). A total difficulties score comprised the sum of the hyperactive, emotional and conduct problem scores. For these scores the higher the score the more problematic the behaviour. In contrast the higher the prosocial score the better the behaviour. There is no published clinical cut-off for these measures.

*The Strengths and Difficulties Questionnaire (SDQ)*

This questionnaire was developed by Robert Goodman from previous versions of behaviour scales such as that of Rutter,^11^ and its later adaption, the Revised Rutter.^12^ It comprises 25 statements and measures prosocial, hyperactive, emotional, conduct and peer relationships, each of which is derived from five items. The 25-items assess different aspects of child’s behaviour in the last 6 months using four response options (‘Not true’, ‘Somewhat true’, ‘Certainly True’, ‘Don’t know’).

The SDQ is well validated^13,14^ and has been shown to emphasise the child’s strengths and weaknesses.^15^ A total behaviour difficulties score is derived from summing the hyperactive, emotional, conduct and peer problems; for each of these scores, the higher the score the worse the behaviour. In contrast, the higher the prosocial score the more desirable the behaviour. In general, the questions were in relation to the past 6 months. They were asked of the chief carer (usually the mother) at ages 47, 81, 115 and 140 months.

In addition, the child’s primary school teacher completed the SDQ at the end of school years 3 and 6 (ages 7-8 and 10-11 years respectively). The behaviour of the study child was recorded on paper questionnaires, one for each child in the class, by the child’s class teacher towards the end of school years 3 and 6. All primary schools in the study area were approached, and for the children who had moved out of the area, parents were sent the questionnaire to give to the teacher.

We use here the prorated scores, derived when there are a few missing variables. However, if all (or almost all) are missing the prorated score is put to missing. Internal consistency across the different constructs of the SDQ and across different informants (self-report, teacher, parent) has been found to be satisfactory (Cronbach's Alpha mean of 0.73). Test–retest stability after 4–6 months has been reported to be 0.62.^16^

*Feeding difficulties*

Questions were asked of the mother when the child was aged 6 years 9 months in regard to the eating characteristics of the child. From the data a scale was derived ranging from 0 to 10.

*The DAWBA*

At the age of 91 months, the Developmental and Well-being Assessment (DAWBA), an extensively described structured questionnaire that involves a mixture of closed questions with fixed response options and [open-ended questions](https://www.sciencedirect.com/topics/psychology/open-ended-question) eliciting free text answers was administered.^17^ The interview's questions are closely related to *DSM-IV* diagnostic criteria and focus on current as opposed to [lifetime](https://www.sciencedirect.com/topics/medicine-and-dentistry/lifespan) problems. The questionnaire was completed by the mothers, and the teachers completed a partial version covering hyperactivity/attention and some aspects of conduct disorder. Scales from these questions were developed and are used here.

***Selective Attention***

Research has strongly indicated that there are functionally and anatomically distinct attention systems, including a system for voluntarily maintaining attention in the absence of strong environmental facilitation and a system for selection. The importance of attentional control systems for the allocation for attention across different tasks simultaneously has also been recognised. A number of attention tasks, developed by Tom Manly and Ian Robertson at the MRC Applied Psychology Unit in Cambridge, UK and Vicki Anderson at the University of Melbourne in Australia, have been designed to show normal variation within a normal population and to distinguish children with attention problems from those without. Their tasks used here, taken from the TEA-Ch, the Tests of Everyday Attention for Children (adapted from the adult version by Robertson^18^) reflect different aspects of attention and appear to measure selective attention, the ability to divide attention between two tasks and attentional control.

The children were tested both at Focus@8 and Focus @ 11

Three tasks were selected from the [TEA-Ch](https://www.sciencedirect.com/topics/medicine-and-dentistry/test-of-everyday-attention), each found to load on a different aspect of attention in factor analytic studies: the Sky search task; the sky search dual task; and the opposite-worlds task. The Sky search task has been found to load on selective attention factors in previous research (e.g. Manly et al.^19^) participants are required to identify pairs of identical “spacecraft” from a page of visually similar stimuli whilst ignoring all distracting stimuli and to circle each pair of identical spaceships. Twenty pairs of space craft were identical from 49 displayed. Time and accuracy were recorded and a [motor control](https://www.sciencedirect.com/topics/medicine-and-dentistry/motor-control) condition was also performed. An age-corrected normative score was calculated based on the manual instructions, which was also adjusted for motor control. The sky search dual task has been found to load on [sustained attention](https://www.sciencedirect.com/topics/psychology/sustained-attention) factors^19^ and followed the same procedure as the sky search task but with the addition of simultaneously presented [auditory stimuli](https://www.sciencedirect.com/topics/medicine-and-dentistry/auditory-stimulation) which participants had to count whilst performing the sky search task. Normative scores based on time and errors were calculated. In previous research,^19^ the opposite-worlds task had loaded on a factor which was labelled attentional control/switching. It involves two conditions: in the first, participants followed digits (1 and 2 only) printed on a hand out stating the number out loud; in the second condition, participants had to inhibit the prepotent response and this time state “one” when presented with the digit 2, and “two” when presented with the digit 1. Errors resulted in a time penalty and normative scores were calculated based on this.

***Sensation seeking***

Sensation seeking has been defined as “the need for varied, novel and complex sensations and experiences and the willingness to take physical and social risks for the sake of such experiences”^20^ and has been applied in relation to potential risk behaviour. The Sensation Seeking questionnaire used at Focus 11+ was a modified version of Arnett’s Inventory of Sensation Seeking (AISS^21^) used to assess risk taking behaviour in the children. This measure has been found to reliably measure both age and sex differences in sensation seeking. The original version of the AISS contains 20 questions, 11 of these were chosen for inclusion at Focus11+ and a further 9 questions designed by Dieter Wolke and Andrea Waylen were incorporated – these were more age appropriate than the original questions.

**References**

1.Wechsler D, Golombok S, Rust J. WISC-CN2I^UK^ Wechsler Intelligence Scale for Children – Third Edition UK Manual. Sidcup, UK: The Psychological Corporation, 1992.

2.Siekierski BM, Jarratt KP, Rosenthal EN, Riccio CA. WISC-III Freedom from Distractibility Index and Measures of Attention in Children. 2003.

3. McCrimmon AW, Smith AD. Review of the Wechsler Abbreviated Scale of Intelligence, (WASI-II). *Journal of Psychoeducational Assessment* 2012; 31(3):337-341. doi.[10.1177/0734282912467756](https://doi.org/10.1177%2F0734282912467756)

4. Wechsler D. Wechsler Abbreviated Scale of Intelligence–Second Edition (WASI-II). San Antonio, Texas: NCS Pearson, 2011.

5. Carey W, McDevitt S. Infant Temperament Questionnaire (4–8 months). Philadelphia: Department Educational Psychology, Temple University, 1977.

6. Carey WB, McDevitt SC. Revision of the Infant Temperament Questionnaire. *Pediatrics* 1978; 61:735–739.

7. Chong SY, Chittleborough CR, Gregory T, Lynch JW, Smithers LG. How many infants are temperamentally difficult? Comparing norms from the Revised Infant Temperament Questionnaire to a population sample of UK infants. *Infant Behavior and Development* 2015; 40*:*20-28.

8. Fullard W, McDevitt SC, Carey WB. Toddler Temperament Scale (1-3 year old children). Dept. Educational Psychology, Temple University, Philadelphia, 1978.

9. Buss AH, Plomin R. Temperament: Early Developing Personality Traits. Hillsdale, New Jersey: Lawrence Erlbaum, 1984.

10. Bornstein MH, Hahn CS, Putnick DL, Pearson R. Stability of child temperament: Multiple moderation by child and mother characteristics. *British Journal of Developmental Psychology* 2019; 37:51-67.

11. Elander J, Rutter M. Use and development of the Rutter parents’ and teachers’ scales. *International Journal of Methods in Psychiatric Research* 1996; 6:63–7.

12. Rutter M. A children's behaviour questionnaire for completion by teachers: preliminary findings. *Child Psychology & Psychiatry & Allied Disciplines* 1967; 8:1-11.

13. Goodman R, Meltzer H, Bailey V. The Strengths and Difficulties Questionnaire: A pilot study on the validity of the self-report version. *European Child and Adolescent Psychiatry* 1998; 7*:*125-130.

14. Goodman R, Scott S. Comparing the Strengths and Difficulties Questionnaire and the Child Behavior Checklist: Is small beautiful? *Journal of Abnormal Child Psychology* 1999; 27: 17-24.

15. Goodman R. The strengths and difficulties questionnaire: A research note. *Journal of Child Psychology and Psychiatry and Allied Disciplines* 1997; 38(5):581-586.

16. Goodman R. Psychometric properties of the strengths and difficulties questionnaire. *Journal of the American Academy of Child and Adolescent Psychiatry* 2001; 40:1337-1345.

17. Goodman R, Ford T, Richards H, Gatward R, Meltzer H. The Development and Well-Being Assessment: Description and initial validation of an integrated assessment of child and adolescent psychopathology. *Journal of Child Psychology and Psychiatry* 2000; 41:645-55.

18. Robertson IH, Ward T, Ridgeway V, Nimmo-Smith I. The structure of normal human attention: The Test of Everyday Attention. *Journal of the International Neuropsychological Society* 1996; 2(6):525-34.

19. Manly T, Anderson V, Nimmo-Smith I, Turner A, Watson P, Robertson IH. The differential assessment of children's attention: the Test of Everyday Attention for Children (TEA-Ch), normative sample and ADHD performance. *Journal of Child Psychology and Psychiatry* 2001; 42:1065-1081

20. Zuckerman M. Sensation seeking*.* Beyond the optimum level of arousal. Hillsdale, New Jersey: Lawrence Erlbaum, 1979.

21. Arnett J. Sensation seeking: A new conceptualisation and a new scale. *Personality and Individual Differences* 1994; 16:289-296.

eTable 1. The variables that were associated with intake of paracetamol at the unadjusted P<0.0001 level; note that two variables were included even though they did not meet this criteria: a history of rheumatism and the social class (based on the partner’s occupation)

| **Variable** | **Univariable analyses** | | |
| --- | --- | --- | --- |
|  | **N** | **OR (95% CI)** | **P** |
| ***Background aspects of health***  Hay fever (d150) | 11163 | 1.229 (1.134, 1.331) | **5.3 x 10^-7^** |
| Asthma (d153) | 11308 | 1.442 (1.285, 1.620) | **5.9 x 10^-10^** |
| Indigestion (d151) | 11186 | 1.593 (1.465, 1.732) | **1.1 x 10^-27^** |
| Eczema (d154) | 11257 | 1.190 (1.090, 1.299) | **1.0 x 10^-4^** |
| Back pain (d158) | 11324 | 1.473 (1.367, 1.587) | **2.9 x 10^-24^** |
| Rheumatism (d162) | 11225 | 1.343 (1.120, 1.610) | **0.001** |
| Pelvic inflammatory disease (d166a) | 11440 | 0.621 (0.488, 0.789) | **9.9 x 10^-5^** |
| Hypertension (d046) | 11217 | 0.723 (0.650, 0.803) | **1.6 x 10^-9^** |
| Migraine (d157) | 11205 | 2.005 (1.858, 2.164) | **7.0 x 10^-72^** |
| ***Health 18-32 weeks***  Pre-pregnancy BMI (dw042) | 10619 | 1.046 (1.035, 1.057) | **4.7 x 10^-18^** |
| In poor health (c050) | 11972 | 1.594 (1.524, 1.666) | **4.2 x 10^-94^** |
| Had a cold (c058) | 11994 | 1.794 (1.666, 1.931) | **3.0 x 10^-54^** |
| Had flu (c059) | 11994 | 2.412 (2.060, 2.824) | **7.0 x 10^-28^** |
| Had an infection (c064) | 11994 | 1.737 (1.600, 1.885) | **8.1 x 10^-40^** |
| Had a headache (c073) | 11994 | 4.733 (4.355, 5.144) | **5.7 x 10^-293^** |
| Had backache (c074) | 11994 | 1.737 (1.580, 1.909) | **2.3 x 10^-30^** |
| Anxiety (c574) | 11853 | 1.071 (1.061, 1.082) | **4.4 x 10^-40^** |
| Depression (c601) | 11971 | 1.052 (1.044, 1.060) | **3.2 x 10^-43^** |
| Malaise (c583) | 11850 | 1.037 (1.032, 1.042) | **1.6 x 10^-56^** |
| ***Maternal lifestyle*** |  |  |  |
| Healthy diet score (c00fac1) | 11962 | 0.818 (0.788, 0.849) | **2.2 x 10^-26^** |
| Processed diet score (c00fac3) | 11962 | 1.131 (1.091, 1.174) | **4.7 x 10^-11^** |
| Ever smoked (b650) | 11685 | 1.312 (1.219, 1.412) | **3.8 x 10^-13^** |
| Number of cigarettes smoked (c483) | 12001 | 1.280 (1.222, 1.341) | **3.9 x 10^-25^** |
| Any passive smoking (c481a) | 10404 | 1.323 (1.221, 1.433) | **7.8 x 10^-12^** |
| No alcohol consumed (b722) | 11447 | 0.706 (0.655, 0.760) | **3.2 x 10^-20^** |
|  |  |  |  |
| ***Social conditions*** |  |  |  |
| Council housing (a006) | 11625 | 1.644 (1.474, 1.834) | **5.3 x 10^-19^** |
| Crowding index (a551) | 11459 | 1.258 (1.209, 1.310) | **5.0 x 10^-29^** |
| Maternal education (c645a) | 11931 | 0.894 (0.869, 0.920) | **1.1 x 10^-14^** |
| Domestic cleaning chemical score (achem) | 11775 | 1.041 (1.033, 1.049) | **2.5 x 10^-24^** |
| Mother worked in pregnancy (c491) | 11347 | 0.690 (0.637, 0.747) | **8.7 x 10^-20^** |
| Social class (c765) | 10845 | 1.012 (1.000, 1.024) | **0.041** |
| Age at first pregnancy (b023) | 11748 | 0.962 (0.955, 0.969) | **3.7 x 10^-24^** |
| Parity (b032) | 11560 | 1.298 (1.248, 1.350) | **1.4 x 10^-38^** |

(In brackets are the variable numbers used in the analysis)

eTable 2. Proportion of paracetamol taking mothers for whom all potential confounders were available.

| **OUTCOME MEASURE** | **NO. WITH**  **PARACETAMOL**  **INFORMATION** | **PROPORTION (N) WITH**  **PARACETAMOL AND ALL**  **CONFOUNDER INFORMATION** |
| --- | --- | --- |
|  |  |  |
| IQ at age 8 | 6621 | 84.0% (5562) |
| IQ at age 15 | 4468 | 84.7% (3783) |
| Temperament at 6m | 9850 | 82.8% (8160) |
| Temperament at 24m | 9655 | 83.3% (8041) |
| SDQ behaviour at 47m | 8975 | 83.7% (7508) |
| SDQ behaviour at 6y | 7969 | 84.4% (6723) |
| SDQ behaviour at 11y | 6675 | 85.1% (5680) |
| SDQT behaviour at 7-8 | 5468 | 79.8% (4362) |
| SDQT behaviour at 10-11 | 6281 | 80.0% (5018) |
| DAWBA at 7y | 7716 | 84.5% (6517) |
| DAWBA – T at 7-8y | 5465 | 80.1% (4376) |
| DAWBA – T at 10-11y | 6273 | 80.0% (5021) |
|  |  |  |

SDQ = Strengths and Difficulties Questionnaire completed by parent;

SDQT = Strengths and Difficulties Questionnaire completed by teacher;

DAWBA = Development and Well-Being Assessment completed by parent;

DAWBA – T = Development and Well-Being Assessment completed by teacher.

eTable 3: Associations between maternal paracetamol intake 18-32 weeks and measures of IQ (In brackets are the variable numbers used in the analysis) – All children

|  | Unadjusted | | | |  | Adjusted^1^ | | | |
| --- | --- | --- | --- | --- | --- | --- | --- | --- | --- |
| Outcome | N | Mean diff (95% CI) | P | R^2^ |  | N | Mean diff (95% CI) | P | R^2^ |
|  |  |  |  |  |  |  |  |  |  |
| Verbal IQ at 8 years (f8ws110) | 6651 | -2.229 (-3.040, -1.417) | 7.5 x 10^-8^ | 0.43% |  | 5588 | -0.748 (-1.660, 0.165) | 0.108 | 13.39% |
| Performance IQ at 8 years (f8ws111) | 6641 | -1.845 (-2.675, -1.015) | 1.3 x 10^-5^ | 0.29% |  | 5578 | -0.666 (-1.632, 0.301) | 0.177 | 6.94% |
| Full scale IQ at 8 years (f8ws112) | 6621 | -2.286 (-3.086, -1.486) | 2.2 x 10^-8^ | 0.47% |  | 5562 | -0.760 (-1.655, 0.135) | 0.096 | 13.67% |
| Verbal comprehension index at 8 (f8ws120) | 6598 | -1.388 (-1.936, -0.839) | 7.3 x 10^-7^ | 0.37% |  | 5546 | -0.445 (-1.061, 0.171) | 0.157 | 13.60% |
| Perceptual organisation index at 8 (f8ws121) | 6264 | -0.862 (-1.402, -0.323) | 0.002 | 0.16% |  | 5267 | -0.066 (-0.695, 0.563) | 0.837 | 6.78% |
| Freedom from distractibility at 8 (f8ws122) | 6472 | -0.697 (-0.993, -0.401) | 3.9 x 10^-6^ | 0.33% |  | 5448 | -0.349 (-0.695, -0.002) | 0.048 | 6.45% |
| Vocabulary IQ at 15 years (fh6277) | 4764 | -1.599 (-2.280, -0.917) | 4.4 x 10^-6^ | 0.44% |  | 4027 | -0.618 (-1.383, 0.147) | 0.113 | 13.65% |
| Matrix reasoning score at 15 years (fh6278) | 4469 | -0.562 (-1.105, -0.018) | 0.043 | 0.09% |  | 3784 | -0.209 (-0.838, 0.420) | 0.515 | 5.54% |
| Total IQ at 15 years (fh6280) | 4468 | -1.642 (-2.414, -0.870) | 3.1 x 10^-5^ | 0.39% |  | 3783 | -0.662 (-1.516, 0.192) | 0.129 | 14.59% |

^1^ Adjusted for mother ever having asthma, indigestion, back pain or migraine, pre-pregnancy BMI, subjective assessment of health in late pregnancy, having a cold, flu, an infection or a headache in late pregnancy, healthy diet score, processed diet score, alcohol consumption in pregnancy, domestic cleaning chemical score and parity.

eTable 3a: Associations between maternal paracetamol intake 18-32 weeks and measures of IQ (In brackets are the variable numbers used in the analysis) – Boys only

|  | Unadjusted | | | |  | Adjusted^1^ | | | |
| --- | --- | --- | --- | --- | --- | --- | --- | --- | --- |
| Outcome | N | Mean diff (95% CI) | P | R^2^ |  | N | Mean diff (95% CI) | P | R^2^ |
|  |  |  |  |  |  |  |  |  |  |
| Verbal IQ at 8 years (f8ws110) | 3311 | -2.023 (-3.229, -0.817) | 0.001 | 0.33% |  | 2801 | -0.780 (-2.140, 0.579) | 0.261 | 13.06% |
| Performance IQ at 8 years (f8ws111) | 3311 | -1.889 (-3.100, -0.679) | 0.002 | 0.22% |  | 2799 | -0.756 (-2.173, 0.661) | 0.295 | 6.43% |
| Full scale IQ at 8 years (f8ws112) | 3295 | -2.172 (-3.350, -0.994) | 3.0 x 10^-4^ | 0.40% |  | 2787 | -0.781 (-2.110, 0.548) | 0.249 | 12.74% |
| Verbal comprehension index at 8 (f8ws120) | 3277 | -1.247 (-2.059, -0.434) | 0.003 | 0.28% |  | 2775 | -0.469 (-1.384, 0.446) | 0.315 | 13.66% |
| Perceptual organisation index at 8 (f8ws121) | 3135 | -0.848 (-1.636, -0.060) | 0.035 | 0.14% |  | 2650 | -0.024 (-0.952, 0.904) | 0.959 | 5.88% |
| Freedom from distractibility at 8 (f8ws122) | 3212 | -0.652 (-1.090, -0.214) | 0.004 | 0.26% |  | 2722 | -0.274 (-0.787, 0.238) | 0.294 | 6.54% |
| Vocabulary IQ at 15 years (fh6277) | 2257 | -1.019 (-2.017, -0.020) | 0.046 | 0.18% |  | 1917 | -0.447 (-1.560, 0.667) | 0.432 | 12.93% |
| Matrix reasoning score at 15 years (fh6278) | 2129 | -0.468 (-1.260, 0.323) | 0.246 | 0.06% |  | 1814 | -0.176 (-1.083, 0.732) | 0.704 | 6.53% |
| Total IQ at 15 years (fh6280) | 2129 | -1.302 (-2.449, -0.155) | 0.026 | 0.23% |  | 1814 | -0.762 (-2.014, 0.491) | 0.233 | 14.60% |

eTable 3b: Associations between maternal paracetamol intake 18-32 weeks and measures of IQ (In brackets are the variable numbers used in the analysis) – Girls only

|  | Unadjusted | | | |  | Adjusted^1^ | | | |
| --- | --- | --- | --- | --- | --- | --- | --- | --- | --- |
| Outcome | N | Mean diff (95% CI) | P | R^2^ |  | N | Mean diff (95% CI) | P | R^2^ |
|  |  |  |  |  |  |  |  |  |  |
| Verbal IQ at 8 years (f8ws110) | 3340 | -2.392 (-3.479, -1.304) | 1.7 x 10^-5^ | 0.55% |  | 2787 | -0.656 (-1.880, 0.567) | 0.293 | 14.21% |
| Performance IQ at 8 years (f8ws111) | 3330 | -1.850 (-2.987, -0.713) | 0.001 | 0.30% |  | 2779 | -0.583 (-1.905, 0.739) | 0.387 | 7.71% |
| Full scale IQ at 8 years (f8ws112) | 3236 | -2.399 (-3.484, -1.313) | 1.5 x 10^-5^ | 0.56% |  | 2775 | -0.701 (-1.910, 0.508) | 0.256 | 14.91% |
| Verbal comprehension index at 8 (f8ws120) | 3321 | -1.507 (-2.246, -0.767) | 6.7 x 10^-5^ | 0.37% |  | 2771 | -0.386 (-1.217, 0.445) | 0.362 | 13.99% |
| Perceptual organisation index at 8 (f8ws121) | 3129 | -0.857 (-1.594, -0.119) | 0.023 | 0.17% |  | 2617 | -0.083 (-0.937, 0.771) | 0.848 | 8.21% |
| Freedom from distractibility at 8 (f8ws122) | 3260 | -0.748 (-1.146, -0.349) | 2.4 x 10^-4^ | 0.41% |  | 2726 | -0.413 (-0.882, 0.056) | 0.084 | 6.85% |
| Vocabulary IQ at 15 years (fh6277) | 2507 | -2.075 (-3.006, -1.144) | 1.3 x 10^-5^ | 0.76% |  | 2110 | -0.688 (-1.742, 0.366) | 0.200 | 15.15% |
| Matrix reasoning score at 15 years (fh6278) | 2340 | -0.613 (-1.362, 0.135) | 0.108 | 0.11% |  | 1970 | -0.228 (-1.105, 0.649) | 0.610 | 5.11% |
| Total IQ at 15 years (fh6280) | 2339 | -1.884 (-2.926, -0.842) | 4.0 x 10^-4^ | 0.54% |  | 1969 | -0.500 (-1.670, 0.669) | 0.401 | 15.16% |

^1^ Adjusted for mother ever having asthma, indigestion, back pain or migraine, pre-pregnancy BMI, subjective assessment of health in late pregnancy, having a cold, flu, an infection or a headache in late pregnancy, healthy diet score, processed diet score, alcohol consumption in pregnancy, domestic cleaning chemical score and parity.

eTable 4: Associations between maternal paracetamol intake 18-32 weeks and measures of childhood temperament (In brackets are the variable numbers used in the analysis) – All children

|  | Unadjusted | | | |  | Adjusted^1^ | | | |
| --- | --- | --- | --- | --- | --- | --- | --- | --- | --- |
|  | N | Mean diff (95% CI) | P | R^2^ |  | N | Mean diff (95% CI) | P | R^2^ |
| Approach score at 6m (kb802c) | 9850 | 0.472 (0.219, 0.725) | 2.6 x 10^-4^ | 0.14% |  | 8160 | 0.223 (-0.086, 0.532) | 0.157 | 0.97% |
| Adaptability score at 6m (kb803c) | 9861 | 0.588 (0.361, 0.815) | 3.7 x 10^-7^ | 0.26% |  | 8166 | 0.328 (0.054, 0.603) | 0.019 | 1.23% |
| Distractability score at 6m (kb807c) | 9857 | 0.411 (0.188, 0.634) | 3.1 x 10^-4^ | 0.13% |  | 8163 | 0.198 (-0.074, 0.470) | 0.153 | 1.08% |
| Threshold score at 6m (kb808c) | 9845 | 0.645 (0.403, 0.887) | 1.7 x 10^-7^ | 0.28% |  | 8156 | 0.039 (-0.252, 0.330) | 0.793 | 2.23% |
| Difficult baby at 6m (kb_dif) | 10616 | 1.716 (0.986, 2.445) | 4.1 x 10^-6^ | 0.20% |  | 8776 | 0.793 (-0.096, 1.682) | 0.080 | 1.32% |
| Adaptability score at 24m (ke803b) | 9663 | 0.487 (0.319, 0.655) | 1.3 x 10^-8^ | 0.33% |  | 8046 | 0.131 (-0.071, 0.333) | 0.204 | 1.72% |
| Intensity score at 24m (ke804b) | 9702 | 0.416 (0.233, 0.599) | 8.2 x 10^-6^ | 0.20% |  | 8073 | 0.043 (-0.178, 0.264) | 0.704 | 1.33% |
| Mood score at 24m (ke805b) | 9712 | 0.657 (0.429, 0.885) | 1.6 x 10^-8^ | 0.33% |  | 8083 | 0.250 (-0.024, 0.524) | 0.074 | 2.09% |
| Persistence score at 24m (ke806b) | 9700 | 0.331 (0.133, 0.528) | 0.001 | 0.11% |  | 8074 | 0.361 (0.122, 0.600) | 0.003 | 1.39% |
| Emotionality score at 38m (kg620b) | 9455 | 0.371 (0.199, 0.543) | 2.4 x 10^-5^ | 0.19% |  | 7876 | 0.168 (-0.039, 0.374) | 0.111 | 1.65% |
| Difficult child at 24m (ke_dif) | 9655 | 2.157 (1.459, 2.855) | 1.4 x 10^-9^ | 0.38% |  | 8041 | 0.509 (-0.335, 1.353) | 0.237 | 2.10% |

^1^ Adjusted for mother ever having asthma, indigestion, back pain or migraine, pre-pregnancy BMI, subjective assessment of health in late pregnancy, having a cold, flu, an infection or a headache in late pregnancy, healthy diet score, processed diet score, alcohol consumption in pregnancy, domestic cleaning chemical score and parity.

eTable 4a: Associations between maternal paracetamol intake 18-32 weeks and measures of childhood temperament (In brackets are the variable numbers used in the analysis) – Boys only

|  | Unadjusted | | | |  | Adjusted^1^ | | | |
| --- | --- | --- | --- | --- | --- | --- | --- | --- | --- |
|  | N | Mean diff (95% CI) | P | R^2^ |  | N | Mean diff (95% CI) | P | R^2^ |
| Approach score at 6m (kb802c) | 5082 | 0.553 (0.209, 0.897) | 0.002 | 0.20% |  | 4231 | 0.441 (0.026, 0.856) | 0.037 | 1.21% |
| Adaptability score at 6m (kb803c) | 5087 | 0.582 (0.270, 0.894) | 2.6 x 10^-4^ | 0.26% |  | 4233 | 0.442 (0.065, 0.818) | 0.021 | 1.48% |
| Distractability score at 6m (kb807c) | 5087 | 0.483 (0.171, 0.795) | 0.002 | 0.18% |  | 4234 | 0.397 (0.019, 0.776) | 0.040 | 1.54% |
| Threshold score at 6m (kb808c) | 5080 | 0.671 (0.334, 1.009) | 9.8 x 10^-5^ | 0.30% |  | 4230 | 0.107 (-0.296, 0.510) | 0.603 | 2.35% |
| Difficult baby at 6m (kb_dif) | 5463 | 1.802 (0.808, 2.797) | 3.9 x 10^-4^ | 0.23% |  | 4537 | 1.348 (0.152, 2.543) | 0.027 | 1.50% |
| Adaptability score at 24m (ke803b) | 4978 | 0.537 (0.303, 0.771) | 7.1 x 10^-6^ | 0.40% |  | 4172 | 0.225 (-0.055, 0.506) | 0.115 | 2.04% |
| Intensity score at 24m (ke804b) | 4997 | 0.308 (0.048, 0.569) | 0.020 | 0.11% |  | 4189 | -0.050 (-0.362, 0.262) | 0.755 | 1.50% |
| Mood score at 24m (ke805b) | 5004 | 0.782 (0.462, 1.103) | 1.8 x 10^-6^ | 0.45% |  | 4194 | 0.368 (-0.015, 0.750) | 0.060 | 2.54% |
| Persistence score at 24m (ke806b) | 4999 | 0.356 (0.080, 0.632) | 0.012 | 0.13% |  | 4191 | 0.362 (0.030, 0.694) | 0.032 | 2.12% |
| Emotionality score at 38m (kg620b) | 4869 | 0.424 (0.190, 0.657) | 3.8 x 10^-4^ | 0.26% |  | 4071 | 0.273 (-0.006, 0.552) | 0.055 | 2.17% |
| Difficult child at 24m (ke_dif) | 4974 | 2.271 (1.291, 3.252) | 5.7 x 10^-6^ | 0.41% |  | 4170 | 0.826 (-0.349, 2.001) | 0.168 | 2.46% |

eTable 4b: Associations between maternal paracetamol intake 18-32 weeks and measures of childhood temperament (In brackets are the variable numbers used in the analysis) – Girls only

|  | Unadjusted | | | |  | Adjusted^1^ | | | |
| --- | --- | --- | --- | --- | --- | --- | --- | --- | --- |
|  | N | Mean diff (95% CI) | P | R^2^ |  | N | Mean diff (95% CI) | P | R^2^ |
| Approach score at6m (kb802c) | 4768 | 0.342 (-0.029, 0.714) | 0.071 | 0.07% |  | 3929 | -0.004 (-0.461, 0.452) | 0.986 | 1.35% |
| Adaptability score at 6m (kb803c) | 4774 | 0.574 (0.245, 0.904) | 0.001 | 0.24% |  | 3933 | 0.215 (-0.185, 0.615) | 0.293 | 1.59% |
| Distractability score at 6m (kb807c) | 4770 | 0.341 (0.022, 0.659) | 0.036 | 0.09% |  | 3929 | 0.033 (-0.357, 0.424) | 0.867 | 1.43% |
| Threshold score at 6m (kb808c) | 4765 | 0.596 (0.250, 0.942) | 0.001 | 0.24% |  | 3926 | -0.040 (-0.460, 0.380) | 0.851 | 2.78% |
| Difficult baby 6m (kb_dif) | 5153 | 1.582 (0.511, 2.652) | 0.004 | 0.16% |  | 4239 | 0.289 (-1.035, 1.613) | 0.669 | 1.57% |
| Adaptability score at 24m (ke803b) | 4685 | 0.463 (0.223, 0.702) | 1.5 x 10^-4^ | 0.31% |  | 3874 | 0.043 (-0.247, 0.334) | 0.770 | 1.76% |
| Intensity score at 24m (ke804b) | 4705 | 0.536 (0.279, 0.792) | 4.3 x 10^-5^ | 0.35% |  | 3884 | 0.149 (-0.165, 0.462) | 0.353 | 1.44% |
| Mood score at 24m (ke805b) | 4708 | 0.533 (0.209, 0.857) | 0.001 | 0.22% |  | 3889 | 0.110 (-0.283, 0.503) | 0.584 | 2.23% |
| Persistence score at 24m (ke806b) | 4701 | 0.333 (0.052, 0.613) | 0.020 | 0.12% |  | 3883 | 0.360 (0.018, 0.703) | 0.039 | 1.44% |
| Emotionality score at 38m (kg620b) | 4586 | 0.293 (0.041, 0.544) | 0.023 | 0.11% |  | 3805 | 0.022 (-0.282, 0.327) | 0.885 | 1.60% |
| Difficult child at 24m (ke_dif) | 4681 | 2.049 (1.054, 3.044) | 5.5 x 10^-5^ | 0.35% |  | 3871 | 0.130 (-1.086, 1.345) | 0.834 | 2.08% |

^1^ Adjusted for mother ever having asthma, indigestion, back pain or migraine, pre-pregnancy BMI, subjective assessment of health in late pregnancy, having a cold, flu, an infection or a headache in late pregnancy, healthy diet score, processed diet score, alcohol consumption in pregnancy, domestic cleaning chemical score and parity.

eTable 5: Associations between maternal paracetamol intake 18-32 weeks and measures of hyperactivity using the SDQ (In brackets are the variable numbers used in the analysis) – All children

|  | Unadjusted | | | |  | Adjusted^1^ | | | |
| --- | --- | --- | --- | --- | --- | --- | --- | --- | --- |
| Hyperactivity score | N | Mean diff (95% CI) | P | R^2^ |  | N | Mean diff (95% CI) | P | R^2^ |
| At 42 months (kj645) | 9422 | 0.232 (0.158, 0.306) | 8.1 x 10^-10^ | 0.40% |  | 7849 | 0.163 (0.074, 0.252) | 3.3 x 10^-4^ | 2.74% |
| At 47 months (j556b) | 8975 | 0.408 (0.311, 0.504) | 1.3 x 10^-16^ | 0.76% |  | 7508 | 0.219 (0.104, 0.333) | 1.8 x 10^-4^ | 5.01% |
| At 81 months (kq346b) | 7975 | 0.303 (0.198, 0.407) | 1.5 x 10^-8^ | 0.40% |  | 6732 | 0.124 (-0.001, 0.249) | 0.051 | 3.02% |
| At 115 months (ku706b) | 7310 | 0.279 (0.174, 0.383) | 1.6 x 10^-7^ | 0.37% |  | 6171 | 0.091 (-0.033, 0.214) | 0.150 | 2.45% |
| At 140 months (kw6601b) | 6665 | 0.256 (0.149, 0.364) | 3.1 x 10^-6^ | 0.33% |  | 5670 | 0.045 (-0.082, 0.171) | 0.489 | 2.68% |
| In school year 3 (age 7/8) (sa162b) | 5469 | 0.279 (0.137, 0.421) | 1.2 x 10^-4^ | 0.27% |  | 4363 | 0.155 (-0.014, 0.325) | 0.072 | 3.14% |
| In school year 6 (age 10/11) (se162b) | 6281 | 0.199 (0.068, 0.331) | 0.003 | 0.14% |  | 5018 | 0.048 (-0.109, 0.205) | 0.551 | 3.33% |

^1^ Adjusted for mother ever having asthma, indigestion, back pain or migraine, pre-pregnancy BMI, subjective assessment of health in late pregnancy, having a cold, flu, an infection or a headache in late pregnancy, healthy diet score, processed diet score, alcohol consumption in pregnancy, domestic cleaning chemical score and parity.

eTable 5a: Associations between maternal paracetamol intake 18-32 weeks and measures of hyperactivity using the SDQ (In brackets are the variable numbers used in the analysis) – Boys only

|  | Unadjusted | | | |  | Adjusted^1^ | | | |
| --- | --- | --- | --- | --- | --- | --- | --- | --- | --- |
| Hyperactivity score | N | Mean diff (95% CI) | P | R^2^ |  | N | Mean diff (95% CI) | P | R^2^ |
| At 42 months (kj645) | 4865 | 0.263 (0.158, 0.369) | 1.1 x 10^-6^ | 0.49% |  | 4068 | 0.166 (0.040, 0.293) | 0.010 | 2.55% |
| At 47 months (j556b) | 4635 | 0.465 (0.328, 0.602) | 3.0 x 10^-11^ | 0.95% |  | 3895 | 0.195 (0.033, 0.357) | 0.019 | 5.55% |
| At 81 months (kq346b) | 4095 | 0.316 (0.164, 0.468) | 4.9 x 10^-5^ | 0.40% |  | 3472 | 0.135 (-0.046, 0.316) | 0.143 | 2.97% |
| At 115 months (ku706b) | 3706 | 0.305 (0.151, 0.460) | 1.1 x 10^-4^ | 0.40% |  | 3146 | 0.100 (-0.084, 0.283) | 0.287 | 2.94% |
| At 140 months (kw6601b) | 3352 | 0.247 (0.087, 0.406) | 0.002 | 0.27% |  | 2864 | 0.055 (-0.132, 0.243) | 0.564 | 2.90% |
| In school year 3 (age 7/8) (sa162b) | 2742 | 0.303 (0.082, 0.523) | 0.007 | 0.26% |  | 2186 | 0.132 (-0.132, 0.396) | 0.328 | 4.52% |
| In school year 6 (age 10/11) (se162b) | 3173 | 0.298 (0.094, 0.502) | 0.004 | 0.26% |  | 2536 | 0.116 (-0.131, 0.363) | 0.356 | 4.49% |

eTable 5b: Associations between maternal paracetamol intake 18-32 weeks and measures of hyperactivity using the SDQ (In brackets are the variable numbers used in the analysis) – Girls only

|  | Unadjusted | | | |  | Adjusted^1^ | | | |
| --- | --- | --- | --- | --- | --- | --- | --- | --- | --- |
| Hyperactivity score | N | Mean diff (95% CI) | P | R^2^ |  | N | Mean diff (95% CI) | P | R^2^ |
| At 42 months (kj645) | 4557 | 0.213 (0.111, 0.316) | 4.6 x 10^-5^ | 0.36% |  | 3781 | 0.173 (0.049, 0.298) | 0.006 | 3.58% |
| At 47 months (j556b) | 4340 | 0.371 (0.237, 0.504) | 5.5 x 10^-8^ | 0.68% |  | 3613 | 0.252 (0.093, 0.411) | 0.002 | 4.98% |
| At 81 months (kq346b) | 3880 | 0.330 (0.191, 0.468) | 3.2 x 10^-6^ | 0.56% |  | 3260 | 0.138 (-0.028, 0.305) | 0.104 | 4.16% |
| At 115 months (ku706b) | 3604 | 0.274 (0.140, 0.409) | 6.6 x 10^-5^ | 0.44% |  | 3025 | 0.088 (-0.072, 0.248) | 0.280 | 2.73% |
| At 140 months (kw6601b) | 3313 | 0.290 (0.152, 0.428) | 3.7 x 10^-5^ | 0.51% |  | 2806 | 0.057 (-0.106, 0.220) | 0.490 | 3.67% |
| In school year 3 (age 7/8) (sa162b) | 2727 | 0.333 (0.172, 0.493) | 4.8 x 10^-5^ | 0.60% |  | 2177 | 0.207 (0.017, 0.396) | 0.033 | 3.06% |
| In school year 6 (age 10/11) (se162b) | 3108 | 0.165 (0.026, 0.304) | 0.020 | 0.17% |  | 2482 | 0.009 (-0.154, 0.171) | 0.918 | 3.21% |

^1^ Adjusted for mother ever having asthma, indigestion, back pain or migraine, pre-pregnancy BMI, subjective assessment of health in late pregnancy, having a cold, flu, an infection or a headache in late pregnancy, healthy diet score, processed diet score, alcohol consumption in pregnancy, domestic cleaning chemical score and parity.

eTable 6: Associations between maternal paracetamol intake 18-32 weeks and measures of hyperactivity using the DAWBA (In brackets are the variable numbers used in the analysis) – All children

|  | Unadjusted | | | |  | Adjusted^1^ | | | |
| --- | --- | --- | --- | --- | --- | --- | --- | --- | --- |
|  | N | Mean diff (95% CI) | P | R^2^ |  | N | Mean diff (95% CI) | P | R^2^ |
| Number of activity symptoms (kr445a) | 7707 | 0.296 (0.174, 0.418) | 2.0 x 10^-6^ | 0.29% |  | 6510 | 0.108 (-0.035, 0.251) | 0.139 | 2.01% |
| No. of severe activity symptoms (kr446a) | 7707 | 0.124 (0.064, 0.183) | 4.8 x 10^-5^ | 0.21% |  | 6510 | 0.038 (-0.031, 0.108) | 0.278 | 1.51% |
| Activity symptoms score (kr447b) | 7677 | 0.418 (0.254, 0.582) | 6.1 x 10^-7^ | 0.32% |  | 6487 | 0.144 (-0.048, 0.337) | 0.141 | 2.20% |
| Number of attention symptoms (kr457a) | 7689 | 0.277 (0.151, 0.403) | 1.7 x 10^-5^ | 0.24% |  | 6497 | 0.127 (-0.021, 0.276) | 0.094 | 1.52% |
| No. of severe attention symptoms (kr458a) | 7689 | 0.116 (0.054, 0.178) | 2.2 x 10^-4^ | 0.18% |  | 6497 | 0.054 (-0.016, 0.125) | 0.132 | 0.76% |
| Attention symptoms score (kr459b) | 7666 | 0.386 (0.218, 0.554) | 6.9 x 10^-6^ | 0.26% |  | 6481 | 0.172 (-0.025, 0.368) | 0.086 | 1.50% |
| No. of attention/activity symptoms (kr460a) | 7716 | 0.575 (0.348, 0.801) | 6.6 x 10^-7^ | 0.32% |  | 6517 | 0.235 (-0.031, 0.500) | 0.083 | 1.98% |
| Attention symptoms score (sa090b) | 5467 | 0.647 (0.363, 0.931) | 7.9 x 10^-6^ | 0.36% |  | 4378 | 0.451 (0.114, 0.787) | 0.009 | 2.68% |
| Activity symptoms score (sa087b) | 5463 | 0.209 (0.018, 0.399) | 0.032 | 0.08% |  | 4374 | 0.067 (-0.155, 0.290) | 0.553 | 2.02% |
| Attention/activity symptoms score (sa093b) | 5465 | 0.859 (0.426, 1.293) | 1.0 x 10^-4^ | 0.28% |  | 4376 | 0.528 (0.018, 1.037) | 0.042 | 2.75% |
| Attention symptoms score (se090b) | 6273 | 0.264 (0.015, 0.512) | 0.037 | 0.07% |  | 5021 | -0.024 (-0.317, 0.268) | 0.871 | 3.02% |
| Activity symptoms score (se087b) | 6268 | 0.176 (-0.001, 0.353) | 0.051 | 0.06% |  | 5015 | -0.048 (-0.255, 0.159) | 0.650 | 2.20% |
| Attention/activity symptoms score (se093b) | 6273 | 0.441 (0.050, 0.831) | 0.027 | 0.08% |  | 5021 | -0.075 (-0.532, 0.382) | 0.747 | 3.11% |

^1^ Adjusted for mother ever having asthma, indigestion, back pain or migraine, pre-pregnancy BMI, subjective assessment of health in late pregnancy, having a cold, flu, an infection or a headache in late pregnancy, healthy diet score, processed diet score, alcohol consumption in pregnancy, domestic cleaning chemical score and parity.

eTable 6a: Associations between maternal paracetamol intake 18-32 weeks and measures of hyperactivity using the DAWBA (In brackets are the variable numbers used in the analysis) – Boys only

|  | Unadjusted | | | |  | Adjusted^1^ | | | |
| --- | --- | --- | --- | --- | --- | --- | --- | --- | --- |
|  | N | Mean diff (95% CI) | P | R^2^ |  | N | Mean diff (95% CI) | P | R^2^ |
| Number of activity symptoms (kr445a) | 3959 | 0.337 (0.152, 0.521) | 3.6 x 10^-4^ | 0.32% |  | 3360 | 0.163 (-0.054, 0.380) | 0.142 | 2.24% |
| No. of severe activity symptoms (kr446a) | 3959 | 0.175 (0.076, 0.274) | 0.001 | 0.30% |  | 3360 | 0.053 (-0.061, 0.168) | 0.361 | 2.09% |
| Activity symptoms score (kr447b) | 3943 | 0.517 (0.260, 0.773) | 7.9 x 10^-5^ | 0.39% |  | 3348 | 0.223 (-0.077, 0.523) | 0.145 | 2.61% |
| Number of attention symptoms (kr457a) | 3943 | 0.262 (0.073, 0.451) | 0.007 | 0.19% |  | 3349 | 0.138 (-0.086, 0.362) | 0.226 | 1.56% |
| No. of severe attention symptoms (kr458a) | 3943 | 0.169 (0.067, 0.271) | 0.001 | 0.27% |  | 3349 | 0.065 (-0.052, 0.181) | 0.278 | 1.07% |
| Attention symptoms score (kr459b) | 3927 | 0.428 (0.167, 0.689) | 0.001 | 0.26% |  | 3338 | 0.202 (-0.102, 0.506) | 0.193 | 1.68% |
| No. of attention/activity symptoms (kr460a) | 3963 | 0.602 (0.260, 0.944) | 0.001 | 0.30% |  | 3363 | 0.296 (-0.106, 0.699) | 0.149 | 2.14% |
| Attention symptoms score (sa090b) | 2744 | 0.536 (0.094, 0.979) | 0.018 | 0.21% |  | 2197 | 0.286 (-0.239, 0.811) | 0.286 | 3.39% |
| Activity symptoms score (sa087b) | 2740 | 0.318 (-0.010, 0.647) | 0.058 | 0.13% |  | 2193 | 0.028 (-0.354, 0.410) | 0.886 | 2.91% |
| Attention/activity symptoms score (sa093b) | 2742 | 0.866 (0.162, 1.569) | 0.016 | 0.21% |  | 2195 | 0.335 (-0.492, 1.161) | 0.427 | 3.58% |
| Attention symptoms score (se090b) | 3169 | 0.477 (0.086, 0.868) | 0.017 | 0.18% |  | 2537 | 0.100 (-0.365, 0.566) | 0.672 | 4.33% |
| Activity symptoms score (se087b) | 3168 | 0.366 (0.065, 0.668) | 0.017 | 0.18% |  | 2534 | -0.028 (-0.384, 0.329) | 0.878 | 3.70% |
| Attention/activity symptoms score (se093b) | 3169 | 0.848 (0.215, 1.481) | 0.009 | 0.22% |  | 2537 | 0.071 (-0.678, 0.820) | 0.852 | 4.73% |

^1^ Adjusted for mother ever having asthma, indigestion, back pain or migraine, pre-pregnancy BMI, subjective assessment of health in late pregnancy, having a cold, flu, an infection or a headache in late pregnancy, healthy diet score, processed diet score, alcohol consumption in pregnancy, domestic cleaning chemical score and parity.

eTable 6b: Associations between maternal paracetamol intake 18-32 weeks and measures of hyperactivity using the DAWBA (In brackets are the variable numbers used in the analysis) – Girls only

|  | Unadjusted | | | |  | Adjusted^1^ | | | |
| --- | --- | --- | --- | --- | --- | --- | --- | --- | --- |
|  | N | Mean diff (95% CI) | P | R^2^ |  | N | Mean diff (95% CI) | P | R^2^ |
| Number of activity symptoms (kr445a) | 3748 | 0.288 (0.134, 0.441) | 2.4 x 10^-4^ | 0.36% |  | 3150 | 0.068 (-0.112, 0.248) | 0.459 | 2.41% |
| No. of severe activity symptoms (kr446a) | 3748 | 0.082 (0.019, 0.145) | 0.011 | 0.17% |  | 3150 | 0.019 (-0.055, 0.093) | 0.609 | 1.24% |
| Activity symptoms score (kr447b) | 3734 | 0.363 (0.166, 0.559) | 3.0 x 10^-4^ | 0.35% |  | 3139 | 0.077 (-0.154, 0.307) | 0.513 | 2.40% |
| Number of attention symptoms (kr457a) | 3746 | 0.331 (0.170, 0.491) | 5.5 x 10^-5^ | 0.43% |  | 3148 | 0.142 (-0.046, 0.330) | 0.140 | 2.47% |
| No. of severe attention symptoms (kr458a) | 3746 | 0.073 (0.007, 0.139) | 0.029 | 0.13% |  | 3148 | 0.046 (-0.031, 0.123) | 0.243 | 0.85% |
| Attention symptoms score (kr459b) | 3739 | 0.393 (0.191, 0.596) | 1.5 x 10^-4^ | 0.39% |  | 3143 | 0.168 (-0.069, 0.406) | 0.164 | 2.21% |
| No. of attention/activity symptoms (kr460a) | 3753 | 0.620 (0.335, 0.904) | 2.0 x 10^-5^ | 0.48% |  | 3154 | 0.211 (-0.120, 0.543) | 0.212 | 2.75% |
| Attention symptoms score (sa090b) | 2723 | 0.896 (0.571, 1.221) | 6.8 x 10^-8^ | 1.06% |  | 2181 | 0.657 (0.272, 1.042) | 0.001 | 3.18% |
| Activity symptoms score (sa087b) | 2723 | 0.193 (0.025, 0.360) | 0.024 | 0.19% |  | 2181 | 0.137 (-0.057, 0.332) | 0.167 | 2.54% |
| Attention/activity symptoms score (sa093b) | 2723 | 1.086 (0.641, 1.530) | 1.7 x 10^-6^ | 0.84% |  | 2181 | 0.793 (0.271, 1.315) | 0.003 | 3.37% |
| Attention symptoms score (se090b) | 3104 | 0.158 (-0.104, 0.420) | 0.237 | 0.05% |  | 2484 | -0.110 (-0.413, 0.193) | 0.478 | 2.44% |
| Activity symptoms score (se087b) | 3100 | 0.054 (-0.099, 0.208) | 0.489 | 0.02% |  | 2481 | -0.052 (-0.224, 0.121) | 0.557 | 1.49% |
| Attention/activity symptoms score (se093b) | 3104 | 0.210 (-0.165, 0.586) | 0.272 | 0.04% |  | 2484 | -0.164 (-0.590, 0.262) | 0.449 | 2.30% |

^1^ Adjusted for mother ever having asthma, indigestion, back pain or migraine, pre-pregnancy BMI, subjective assessment of health in late pregnancy, having a cold, flu, an infection or a headache in late pregnancy, healthy diet score, processed diet score, alcohol consumption in pregnancy, domestic cleaning chemical score and parity.

eTable 7: Associations between maternal paracetamol intake 18-32 weeks and measures of Conduct problems from SDQ and DAWBA (In brackets are the variable numbers used in the analysis) – All children

|  | Unadjusted | | | |  | Adjusted^1^ | | | |
| --- | --- | --- | --- | --- | --- | --- | --- | --- | --- |
| Conduct problems score | N | Mean diff (95% CI) | P | R^2^ |  | N | Mean diff (95% CI) | P | R^2^ |
| At 42 months (kj644) | 9422 | 0.479 (0.384, 0.575) | 1.1 x 10^-22^ | 1.01% |  | 7849 | 0.218 (0.104, 0.333) | 1.9 x 10^-4^ | 3.86% |
| At 47 months (j556d) | 8975 | 0.226 (0.168, 0.285) | 3.9 x 10^-14^ | 0.64% |  | 7508 | 0.081 (0.011, 0.151) | 0.023 | 3.25% |
| At 81 months (kq346d) | 7994 | 0.228 (0.164, 0.293) | 5.3 x 10^-12^ | 0.59% |  | 6742 | 0.101 (0.024, 0.179) | 0.010 | 2.29% |
| At 115 months (ku708b) | 7306 | 0.183 (0.118, 0.248) | 4.2 x 10^-8^ | 0.41% |  | 6169 | 0.072 (-0.006, 0.149) | 0.069 | 2.07% |
| At 140 months (kw6603b) | 6675 | 0.189 (0.121, 0.258) | 6.7 x 10^-8^ | 0.44% |  | 5679 | 0.065 (-0.015, 0.145) | 0.113 | 2.61% |
| In school year 3 (age 7/8) (sa164b) | 5463 | 0.095 (0.020, 0.170) | 0.013 | 0.11% |  | 4358 | 0.033 (-0.054, 0.120) | 0.457 | 2.04% |
| In school year 6 (age 10/11) (se164b) | 6277 | 0.086 (0.007, 0.166) | 0.033 | 0.07% |  | 5015 | -0.032 (-0.123, 0.059) | 0.492 | 2.99% |
| Awkward behaviours score at 7y (kr492b) | 7646 | 0.292 (0.163, 0.420) | 8.7 x 10^-6^ | 0.26% |  | 6467 | 0.059 (-0.093, 0.211) | 0.445 | 1.33% |
| Troublesome behaviours score at 7y (kr519b) | 7666 | 0.135 (0.088, 0.182) | 2.2 x 10^-8^ | 0.41% |  | 6480 | 0.015 (-0.040, 0.071) | 0.589 | 2.05% |
|  |  |  |  |  |  |  |  |  |  |

^1^ Adjusted for mother ever having asthma, indigestion, back pain or migraine, pre-pregnancy BMI, subjective assessment of health in late pregnancy, having a cold, flu, an infection or a headache in late pregnancy, healthy diet score, processed diet score, alcohol consumption in pregnancy, domestic cleaning chemical score and parity.

eTable 7a: Associations between maternal paracetamol intake 18-32 weeks and measures of Conduct problems from SDQ and DAWBA (In brackets are the variable numbers used in the analysis) – Boys only

|  | Unadjusted | | | |  | Adjusted^1^ | | | |
| --- | --- | --- | --- | --- | --- | --- | --- | --- | --- |
| Conduct problems score | N | Mean diff (95% CI) | P | R^2^ |  | N | Mean diff (95% CI) | P | R^2^ |
| At 42 months (kj644) | 4865 | 0.563 (0.426, 0.701) | 1.2 x 10^-15^ | 1.31% |  | 4068 | 0.286 (0.121, 0.450) | 0.001 | 4.62% |
| At 47 months (j556d) | 4635 | 0.261 (0.177, 0.344) | 9.2 x 10^-10^ | 0.81% |  | 3895 | 0.100 (0.001, 0.198) | 0.047 | 4.22% |
| At 81 months (kq346d) | 4104 | 0.251 (0.158, 0.343) | 1.1 x 10^-7^ | 0.68% |  | 3476 | 0.123 (0.014, 0.232) | 0.027 | 2.90% |
| At 115 months (ku708b) | 3701 | 0.244 (0.148, 0.340) | 6.9 x 10^-7^ | 0.66% |  | 3142 | 0.121 (0.006, 0.235) | 0.039 | 3.36% |
| At 140 months (kw6603b) | 3354 | 0.220 (0.117, 0.323) | 2.7 x 10^-5^ | 0.52% |  | 2866 | 0.106 (-0.013, 0.226) | 0.081 | 3.10% |
| In school year 3 (age 7/8) (sa164b) | 2738 | 0.096 (-0.029, 0.222) | 0.133 | 0.08% |  | 2183 | 0.001 (-0.145, 0.147) | 0.992 | 3.32% |
| In school year 6 (age 10/11) (se164b) | 3171 | 0.160 (0.027, 0.293) | 0.018 | 0.18% |  | 2535 | -0.006 (-0.159, 0.147) | 0.935 | 4.78% |
| Awkward behaviours score at 7y (kr492b) | 3927 | 0.406 (0.208, 0.603) | 5.9 x 10^-5^ | 0.41% |  | 3336 | 0.172 (-0.062, 0.405) | 0.149 | 2.00% |
| Troublesome behaviours score at 7y (kr519b) | 3944 | 0.132 (0.062, 0.202) | 2.1 x 10^-4^ | 0.35% |  | 3349 | 0.014 (-0.068, 0.096) | 0.740 | 2.18% |
|  |  |  |  |  |  |  |  |  |  |

eTable 7b: Associations between maternal paracetamol intake 18-32 weeks and measures of Conduct problems from SDQ and DAWBA (In brackets are the variable numbers used in the analysis) – Girls only

|  | Unadjusted | | | |  | Adjusted^1^ | | | |
| --- | --- | --- | --- | --- | --- | --- | --- | --- | --- |
| Conduct problems score | N | Mean diff (95% CI) | P | R^2^ |  | N | Mean diff (95% CI) | P | R^2^ |
| At 42 months (kj644) | 4557 | 0.409 (0.278, 0.541) | 1.0 x 10^-9^ | 0.82% |  | 3781 | 0.157 (-0.0002, 0.315) | 0.050 | 3.46% |
| At 47 months (j556d) | 4340 | 0.196 (0.114, 0.278) | 3.0 x 10^-6^ | 0.50% |  | 3613 | 0.056 (-0.044, 0.155) | 0.273 | 2.72% |
| At 81 months (kq346d) | 3890 | 0.216 (0.125, 0.306) | 3.0 x 10^-6^ | 0.56% |  | 3266 | 0.077 (-0.032, 0.186) | 0.169 | 2.13% |
| At 115 months (ku708b) | 3605 | 0.125 (0.037, 0.213) | 0.005 | 0.53% |  | 3027 | 0.019 (-0.086, 0.124) | 0.722 | 2.01% |
| At 140 months (kw6603b) | 3321 | 0.162 (0.071, 0.253) | 4.7 x 10^-4^ | 0.37% |  | 2813 | 0.015 (-0.092, 0.122) | 0.782 | 3.13% |
| In school year 3 (age 7/8) (sa164b) | 2725 | 0.123 (0.046, 0.200) | 0.002 | 0.36% |  | 2175 | 0.080 (-0.008, 0.169) | 0.075 | 1.69% |
| In school year 6 (age 10/11) (se164b) | 3106 | 0.039 (-0.037, 0.116) | 0.313 | 0.03% |  | 2480 | -0.055 (-0.142, 0.032) | 0.214 | 1.71% |
| Awkward behaviours score at 7y (kr492b) | 3719 | 0.193 (0.031, 0.354) | 0.019 | 0.15% |  | 3131 | -0.057 (-0.249, 0.135) | 0.560 | 1.11% |
| Troublesome behaviours score at 7y (kr519b) | 3722 | 0.142 (0.079, 0.205) | 9.5 x 10^-6^ | 0.53% |  | 3131 | 0.016 (-0.057, 0.090) | 0.669 | 2.54% |
|  |  |  |  |  |  |  |  |  |  |

^1^ Adjusted for mother ever having asthma, indigestion, back pain or migraine, pre-pregnancy BMI, subjective assessment of health in late pregnancy, having a cold, flu, an infection or a headache in late pregnancy, healthy diet score, processed diet score, alcohol consumption in pregnancy, domestic cleaning chemical score and parity.

eTable 8: Associations between maternal paracetamol intake 18-32 weeks and measures of Emotional difficulties using SDQ (In brackets are the variable numbers used in the analysis) – All children

|  | Unadjusted | | | |  | Adjusted^1^ | | | |
| --- | --- | --- | --- | --- | --- | --- | --- | --- | --- |
| Emotional difficulties score | N | Mean diff (95% CI) | P | R^2^ |  | N | Mean diff (95% CI) | P | R^2^ |
| At 42 months (kj643) | 9422 | 0.130 (0.059, 0.201) | 3.5 x 10^-4^ | 0.14% |  | 7864 | 0.042 (-0.043, 0.127) | 0.332 | 2.68% |
| At 47 months (j556c) | 8974 | 0.082 (0.020, 0.145) | 0.010 | 0.07% |  | 7520 | -0.010 (-0.085, 0.065) | 0.798 | 2.06% |
| At 81 months (kq346c) | 7985 | 0.135 (0.061, 0.209) | 3.4 x 10^-4^ | 0.16% |  | 6747 | 0.015 (-0.073, 0.103) | 0.732 | 2.44% |
| At 115 months (ku707b) | 7293 | 0.134 (0.052, 0.215) | 0.001 | 0.14% |  | 6170 | 0.009 (-0.087, 0.105) | 0.852 | 1.69% |
| At 140 months (kw6602b) | 6662 | 0.199 (0.115, 0.282) | 3.4 x 10^-6^ | 0.32% |  | 5682 | 0.013 (-0.087, 0.112) | 0.805 | 2.87% |
| In school year 3 (age 7/8) (sa163b) | 5468 | 0.099 (-0.005, 0.202) | 0.061 | 0.06% |  | 4376 | 0.014 (-0.110, 0.137) | 0.826 | 1.28% |
| In school year 6 (age 10/11) (se163b) | 6280 | 0.111 (0.017, 0.205) | 0.021 | 0.08% |  | 5026 | 0.040 (-0.074, 0.154) | 0.495 | 1.12% |

^1^ Adjusted for mother ever having asthma, indigestion, back pain or migraine, pre-pregnancy BMI, subjective assessment of health in late pregnancy, having a cold, flu, an infection or a headache in late pregnancy, healthy diet score, processed diet score, alcohol consumption in pregnancy, domestic cleaning chemical score and parity.

eTable 8a: Associations between maternal paracetamol intake 18-32 weeks and measures of Emotional difficulties using SDQ (In brackets are the variable numbers used in the analysis) – Boys only

|  | Unadjusted | | | |  | Adjusted^1^ | | | |
| --- | --- | --- | --- | --- | --- | --- | --- | --- | --- |
| Emotional difficulties score | N | Mean diff (95% CI) | P | R^2^ |  | N | Mean diff (95% CI) | P | R^2^ |
| At 42 months (kj643) | 4865 | 0.157 (0.057, 0.257) | 0.002 | 0.19% |  | 4076 | 0.049 (-0.070, 0.168) | 0.421 | 2.57% |
| At 47 months (j556c) | 4634 | 0.131 (0.042, 0.219) | 0.004 | 0.18% |  | 3901 | 0.050 (-0.056, 0.157) | 0.351 | 2.19% |
| At 81 months (kq346c) | 4101 | 0.165 (0.061, 0.268) | 0.002 | 0.24% |  | 3478 | 0.084 (-0.039, 0.206) | 0.182 | 2.29% |
| At 115 months (ku707b) | 3692 | 0.072 (-0.039, 0.182) | 0.205 | 0.04% |  | 3139 | -0.050 (-0.181, 0.081) | 0.456 | 1.50% |
| At 140 months (kw6602b) | 3348 | 0.221 (0.107, 0.335) | 1.5 x 10^-4^ | 0.43% |  | 2867 | 0.061 (-0.075, 0.197) | 0.380 | 2.81% |
| In school year 3 (age 7/8) (sa163b) | 2741 | 0.030 (-0.113, 0.173) | 0.681 | 0.01% |  | 2193 | -0.034 (-0.206, 0.138) | 0.700 | 1.43% |
| In school year 6 (age 10/11) (se163b) | 3172 | 0.091 (-0.041, 0.223) | 0.178 | 0.06% |  | 2539 | 0.052 (-0.109, 0.213) | 0.527 | 1.91% |

eTable 8b: Associations between maternal paracetamol intake 18-32 weeks and measures of Emotional difficulties using SDQ (In brackets are the variable numbers used in the analysis) – Girls only

|  | Unadjusted | | | |  | Adjusted^1^ | | | |
| --- | --- | --- | --- | --- | --- | --- | --- | --- | --- |
| Emotional difficulties score | N | Mean diff (95% CI) | P | R^2^ |  | N | Mean diff (95% CI) | P | R^2^ |
| At 42 months (kj643) | 4557 | 0.097 (-0.003, 0.198) | 0.058 | 0.08% |  | 3788 | 0.034 (-0.087, 0.155) | 0.584 | 3.00% |
| At 47 months (j556c) | 4340 | 0.030 (-0.060, 0.119) | 0.514 | 0.01% |  | 3619 | -0.072 (-0.179, 0.034) | 0.184 | 2.09% |
| At 81 months (kq346c) | 3884 | 0.098 (-0.008, 0.205) | 0.069 | 0.08% |  | 3269 | -0.063 (-0.190, 0.063) | 0.326 | 2.87% |
| At 115 months (ku707b) | 3601 | 0.189 (0.070, 0.309) | 0.002 | 0.27% |  | 3031 | 0.064 (-0.076, 0.203) | 0.372 | 2.42% |
| At 140 months (kw6602b) | 3314 | 0.169 (0.047, 0.291) | 0.007 | 0.22% |  | 2815 | -0.037 (-0.181, 0.107) | 0.613 | 3.36% |
| In school year 3 (age 7/8) (sa163b) | 2727 | 0.161 (0.011, 0.310) | 0.035 | 0.16% |  | 2183 | 0.060 (-0.118, 0.238) | 0.509 | 1.66% |
| In school year 6 (age 10/11) (se163b) | 3108 | 0.132 (-0.002, 0.267) | 0.054 | 0.12% |  | 2487 | 0.025 (-0.136, 0.186) | 0.762 | 1.24% |

^1^ Adjusted for mother ever having asthma, indigestion, back pain or migraine, pre-pregnancy BMI, subjective assessment of health in late pregnancy, having a cold, flu, an infection or a headache in late pregnancy, healthy diet score, processed diet score, alcohol consumption in pregnancy, domestic cleaning chemical score and parity.

eTable 9: Associations between maternal paracetamol intake 18-32 weeks and measures of Total behavioural difficulties using SDQ (In brackets are the variable numbers used in the analysis) – All children

|  | Unadjusted | | | |  | Adjusted^1^ | | | |
| --- | --- | --- | --- | --- | --- | --- | --- | --- | --- |
| Total behavioural difficulties score | N | Mean diff (95% CI) | P | R^2^ |  | N | Mean diff (95% CI) | P | R^2^ |
| At 42 months (kj647) | 9422 | 1.074 (0.842, 1.306) | 1.3 x 10^-19^ | 0.87% |  | 7849 | 0.540 (0.264, 0.816) | 1.3 x 10^-4^ | 4.60% |
| At 47 months (j556f) | 8975 | 0.811 (0.622, 1.001) | 5.8 x 10^-17^ | 0.78% |  | 7508 | 0.307 (0.084, 0.530) | 0.007 | 5.96% |
| At 81 months (kq346f) | 7969 | 0.740 (0.529, 0.950) | 5.9 x 10^-12^ | 0.59% |  | 6723 | 0.244 (-0.004, 0.492) | 0.054 | 3.98% |
| At 115 months (ku710b) | 7282 | 0.732 (0.504, 0.959) | 2.9 x 10^-10^ | 0.54% |  | 6152 | 0.235 (-0.030, 0.501) | 0.082 | 3.51% |
| At 140 months (kw6605b) | 6675 | 0.739 (0.500, 0.977) | 1.4 x 10^-9^ | 0.55% |  | 5680 | 0.120 (-0.158, 0.398) | 0.398 | 4.26% |
| In school year 3 (age 7/8) (sa166b) | 5468 | 0.506 (0.205, 0.806) | 0.001 | 0.20% |  | 4362 | 0.199 (-0.153, 0.551) | 0.268 | 2.91% |
| In school year 6 (age 10/11) (se166b) | 6281 | 0.482 (0.191, 0.773) | 0.001 | 0.17% |  | 5018 | 0.077 (-0.267, 0.420) | 0.661 | 3.16% |

^1^ Adjusted for mother ever having asthma, indigestion, back pain or migraine, pre-pregnancy BMI, subjective assessment of health in late pregnancy, having a cold, flu, an infection or a headache in late pregnancy, healthy diet score, processed diet score, alcohol consumption in pregnancy, domestic cleaning chemical score and parity.

eTable 9a: Associations between maternal paracetamol intake 18-32 weeks and measures of Total behavioural difficulties using SDQ (In brackets are the variable numbers used in the analysis) – Boys only

|  | Unadjusted | | | |  | Adjusted^1^ | | | |
| --- | --- | --- | --- | --- | --- | --- | --- | --- | --- |
| Total behavioural difficulties score | N | Mean diff (95% CI) | P | R^2^ |  | N | Mean diff (95% CI) | P | R^2^ |
| At 42 months (kj647) | 4865 | 1.255 (0.924, 1.587) | 1.4 x 10^-13^ | 1.12% |  | 4068 | 0.605 (0.212, 0.997) | 0.003 | 4.82% |
| At 47 months (j556f) | 4635 | 0.959 (0.689, 1.230) | 4.2 x 10^-12^ | 1.03% |  | 3895 | 0.349 (0.032, 0.667) | 0.031 | 6.88% |
| At 81 months (kq346f) | 4096 | 0.823 (0.517, 1.129) | 1.4 x 10^-7^ | 0.67% |  | 3470 | 0.368 (0.009, 0.726) | 0.044 | 3.96% |
| At 115 months (ku710b) | 3689 | 0.728 (0.388, 1.067) | 2.7 x 10^-5^ | 0.48% |  | 3131 | 0.224 (-0.175, 0.622) | 0.271 | 3.74% |
| At 140 months (kw6605b) | 3356 | 0.782 (0.429, 1.135) | 1.4 x 10^-5^ | 0.56% |  | 2868 | 0.262 (-0.148, 0.672) | 0.210 | 4.24% |
| In school year 3 (age 7/8) (sa166b) | 2741 | 0.435 (-0.031, 0.900) | 0.067 | 0.12% |  | 2185 | 0.059 (-0.486, 0.605) | 0.831 | 3.71% |
| In school year 6 (age 10/11) (se166b) | 3173 | 0.666 (0.219, 1.113) | 0.004 | 0.27% |  | 2536 | 0.236 (-0.294, 0.766) | 0.382 | 4.55% |

eTable 9b: Associations between maternal paracetamol intake 18-32 weeks and measures of Total behavioural difficulties using SDQ (In brackets are the variable numbers used in the analysis) – Girls only

|  | Unadjusted | | | |  | Adjusted^1^ | | | |
| --- | --- | --- | --- | --- | --- | --- | --- | --- | --- |
| Total behavioural difficulties score | N | Mean diff (95% CI) | P | R^2^ |  | N | Mean diff (95% CI) | P | R^2^ |
| At 42 months (kj647) | 4557 | 0.920 (0.599, 1.241) | 2.1 x 10^-8^ | 0.69% |  | 3781 | 0.493 (0.107, 0.879) | 0.012 | 4.74% |
| At 47 months (j556f) | 4340 | 0.692 (0.430, 0.954) | 2.4 x 10^-7^ | 0.61% |  | 3613 | 0.261 (-0.050, 0.571) | 0.100 | 5.43% |
| At 81 months (kq346f) | 3873 | 0.706 (0.423, 0.990) | 1.1 x 10^-6^ | 0.61% |  | 3253 | 0.127 (-0.212, 0.465) | 0.463 | 4.83% |
| At 115 months (ku710b) | 3593 | 0.761 (0.462, 1.060) | 6.3 x 10^-7^ | 0.69% |  | 3021 | 0.241 (-0.107, 0.590) | 0.175 | 4.00% |
| At 140 months (kw6605b) | 3319 | 0.721 (0.402, 1.040) | 9.4 x 10^-6^ | 0.59% |  | 2812 | -0.009 (-0.383, 0.364) | 0.960 | 5.30% |
| In school year 3 (age 7/8) (sa166b) | 2727 | 0.695 (0.334, 1.056) | 1.6 x 10^-4^ | 0.52% |  | 2177 | 0.376 (-0.045, 0.797) | 0.080 | 2.98% |
| In school year 6 (age 10/11) (se166b) | 3108 | 0.404 (0.063, 0.745) | 0.020 | 0.17% |  | 2482 | -0.055 (-0.454, 0.344) | 0.788 | 2.65% |

^1^ Adjusted for mother ever having asthma, indigestion, back pain or migraine, pre-pregnancy BMI, subjective assessment of health in late pregnancy, having a cold, flu, an infection or a headache in late pregnancy, healthy diet score, processed diet score, alcohol consumption in pregnancy, domestic cleaning chemical score and parity.
